# *Symbiodinium*-Induced Formation of Microbialites: Mechanistic Insights From *in Vitro* Experiments and the Prospect of Its Occurrence in Nature

**DOI:** 10.3389/fmicb.2018.00998

**Published:** 2018-05-17

**Authors:** Jörg C. Frommlet, Daniel Wangpraseurt, Maria L. Sousa, Bárbara Guimarães, Mariana Medeiros da Silva, Michael Kühl, João Serôdio

**Affiliations:** ^1^Department of Biology and Centre for Environmental and Marine Studies (CESAM), University of Aveiro, Aveiro, Portugal; ^2^Marine Biological Section, Department of Biology, University of Copenhagen, Helsingør, Denmark; ^3^Department of Chemistry, University of Cambridge, Cambridge, United Kingdom; ^4^Coral Reef and Global Changes Research Group (RECOR), Department of Oceanography, Institute of Geosciences, Federal University of Bahia (UFBA), Salvador, Brazil; ^5^Climate Change Cluster, University of Technology Sydney, Sydney, NSW, Australia

**Keywords:** *Symbiodinium*, coral endosymbiont, free-living life style, bacterial-algal calcification, photosynthesis-induced calcification, microbialite, endolithic algae

## Abstract

Dinoflagellates in the genus *Symbiodinium* exhibit a variety of life styles, ranging from mutualistic endosymbioses with animal and protist hosts to free-living life styles. In culture, *Symbiodinium* spp. and naturally associated bacteria are known to form calcifying biofilms that produce so-called symbiolites, i.e., aragonitic microbialites that incorporate *Symbiodinium* as endolithic cells. In this study, we investigated (i) how algal growth and the combined physiological activity of these bacterial-algal associations affect the physicochemical macroenvironment in culture and the microenvironment within bacterial-algal biofilms, and (ii) how these interactions induce the formation of symbiolites. In batch culture, calcification typically commenced when *Symbiodinium* spp. growth approached stationary phase and when photosynthetic activity and its influence on pH and the carbonate system of the culture medium had already subsided, indicating that symbiolite formation is not simply a function of photosynthetic activity in the bulk medium. Physical disturbance of bacteria-algal biofilms, via repeated detaching and dispersing of the developing biofilm, generally impeded symbiolite formation, suggesting that the structural integrity of biofilms plays an important role in generating conditions conducive to calcification. Microsensor measurements of pH and O_2_ revealed a biofilm microenvironment characterized by high photosynthetic rates and by dynamic changes in photosynthesis and respiration with light intensity and culture age. Ca^2+^ microsensor measurements confirmed the significance of the biofilm microenvironment in inducing calcification, as photosynthesis within the biofilm induced calcification without the influence of batch culture medium and under environmentally relevant flow conditions. Furthermore, first quantitative data on calcification from 26 calcifying cultures enabled a first broad comparison of *Symbiodinium*-induced bacterial-algal calcification with other calcification processes. Our findings support the idea that symbiolite formation is a typical, photosynthesis-induced, bacterial-algal calcification process that is likely to occur under natural conditions.

## Introduction

Dinoflagellates in the genus *Symbiodinium* are important primary producers that engage in trophic endosymbioses with corals and various other animal, and protist hosts (Trench, 1993; Stat et al., 2006). These mutualistic symbioses are of critical importance for tropical coral reef ecosystems, where symbiotic *Symbiodinium* spp., also known as zooxanthellae, provide their hosts with photosynthates in exchange for inorganic nutrients (e.g., Yellowlees et al., 2008). In doing so, they contribute significantly to the coral reef food web (reviewed in Silveira et al., 2017) and they enhance coral calcification (reviewed in Tambutté et al., 2011), demonstrating that *Symbiodinium* spp. both lay the trophic foundation of coral reef ecosystems and play an important role in the formation of the physical reef structure.

Members of the *Symbiodinium* genus, despite their distinctive endosymbiotic life histories, also exhibit temporarily, and probably also exclusively free-living life styles (e.g., Coffroth et al., 2006; Thornhill et al., 2017). The existence of temporarily free-living *Symbiodinium* populations has long been inferred, as the majority of coral species rely on symbiont acquisition from the environment (reviewed in Baird et al., 2009). Besides, the numerous symbiotic strains that were brought into culture over the years demonstrate that at least certain *Symbiodinium* phylotypes do not rely on their hosts as obligate partners (e.g., Schoenberg and Trench, 1980; Trench, 1981; Santos et al., 2001). Field-based studies repeatedly discovered and characterized free-living *Symbiodinium* communities in planktonic, epiphytic, and benthic habitats (e.g., Koike et al., 2007; Venera-Ponton et al., 2010; Granados-Cifuentes et al., 2015). Especially reef sediments appear to be hotspots for the occurrence of free-living *Symbiodinium* spp. (e.g., Littman et al., 2008; Takabayashi et al., 2012; Yamashita and Koike, 2013), and experimental data suggest that reef sediments play an important role as recruitment reservoir for the establishment of host-symbiont associations (Adams et al., 2009; Nitschke et al., 2016). Moreover, environmental *Symbiodinium* populations are increasingly being recognized for their potential to influence the resilience of coral reef ecosystems to stress and their adaptability to environmental change and yet, the life histories of free-living *Symbiodinium* spp. and their ecological niche(s) in the environment remain practically unexplored (Pochon et al., 2014; Boulotte et al., 2016; Suggett et al., 2017; Thornhill et al., 2017).

Sediment-associated *Symbiodinium* populations are presumed to behave much like *Symbiodinium* in culture: diurnally switching between nonmotile and motile stages (Manning and Gates, 2008). A first indication that benthic *Symbiodinium* life histories could be more complex was recently provided by the discovery that a diverse range of cultured *Symbiodinium* strains and their inherent bacterial communities commonly form calcifying bacterial-algal communities (Frommlet et al., 2015a,b). Jointly, these novel calcifying associations produce aragonitic microbialites, termed symbiolites, which encase adjacent *Symbiodinium* as endolithic cells. Symbiolite formation is an autoendolithic process because cells become endolithic as a result of mineral precipitation (Marlow et al., 2015). In the course of symbiolite formation/calcification (the two terms are used synonymously in this study) thin ducts, starting from the endolithic cells and reaching to the symbiolite surface, remain un-calcified (Frommlet et al., 2015a). Probably assisted by these residual connections to the outside environment, endolithic *Symbiodinium* cells remain alive and photosynthetically active for days to weeks, and upon medium exchange they can return to a free-living state by vacating the symbiolite through the described ducts (Frommlet et al., 2015a). Because of this reversibility of the autoendolithic process, symbiolite formation does not appear to be a dead end for endolithic cells but instead points toward a transient endolithic phase in the life history of benthic *Symbiodinium* populations.

Mineralized structures, ranging from simple sheaths and microbialites to complex shells and skeletons, form an integral part of many lifeforms (Riding, 2000; Knoll, 2003). Their formation can be influenced, induced, or strictly controlled by biological activity (Mann, 2001; Weiner and Dove, 2003; Dupraz et al., 2009). Biologically induced mineralization refers to processes that depend on metabolic activity to induce conditions for mineral precipitation. Often, these conditions are induced by the collective physiological activity of a microbial community, then also referred to as “microbially induced mineralization” (Arp et al., 2001; Aloisi, 2008; Gallagher et al., 2012). One of the most prominent microbially induced mineralization processes throughout much of Earth's history is carbonate precipitation. Modern examples include calcifying microbial mats, ooids, oncoids, stromatolite biofilms, and coral reef sediments (e.g., Garcia-Pichel et al., 2004; Ludwig et al., 2005; Werner et al., 2008; Arp et al., 2010). Calcification in these communities is induced by processes such as ammonium oxidation, sulfate reduction, and especially oxygenic photosynthesis, which all have the potential to increase the calcium carbonate saturation state (Ω) to a point where carbonate precipitation occurs from solution (Visscher and Stolz, 2005; Dupraz et al., 2009; Shiraishi, 2012). This influence of microbial activity on Ω and calcification is known as the “intrinsic alkalinity engine” (Dupraz et al., 2009; Gallagher et al., 2012). In its simplest form, oxygenic photosynthesis induces calcification by increasing pH in the surrounding medium through the assimilation of CO_2_ and ${\text{HCO}}_{3}^{-}$ and the release of OH^−^ from carbon concentrating mechanisms, which shifts the carbonate equilibrium toward ${\text{CO}}_{3}^{2-}$ and in turn increases Ω and leads to what is called “*Cis*”-calcification or “photosynthesis-induced carbonate precipitation” (PCP) (McConnaughey and Whelan, 1997; Riding, 2006; Shiraishi, 2012). Besides alkalinity engines such as PCP, microbially induced calcification also crucially depends on the chemical composition and structure of extracellular polymeric substances (EPS), which (i) form a protective and adhesive organic matrix around the cells (Flemming and Wingender, 2010), (ii) create a diffusion-controlled microenvironment, which is strongly influenced by microbial metabolisms, and (iii) provide nucleation points for mineral precipitation (Kawaguchi and Decho, 2002; Dupraz et al., 2009; Decho, 2010).

PCP is attributed predominantly to cyanobacteria and in some cases to diatoms, green and red algae (Awramik and Riding, 1988; Reid et al., 2000; Saghaï et al., 2015). The presence of dinoflagellates in calcifying reef sediments has previously been inferred from marker pigments (Werner et al., 2008; Schoon et al., 2010) but only the discovery of symbiolites provided first direct evidence for PCP by a dinoflagellate (Frommlet et al., 2015a). The latter study showed that symbiolite growth is light dependent, indicating that the underlying calcification process is induced by the photosynthetic activity of *Symbiodinium*, and that bacteria are essential for the process because (i) symbiolite formation was fully inhibited by antibiotics, and (ii) symbiolite formation could be reinitiated by inoculating previously antibiotics-treated *Symbiodinium* cultures with bacteria from a calcifying *Symbiodinium* culture. The study also demonstrated co-localization of symbiolites with acidic polysaccharides, a group of polysaccharides with important Ca^2+^-binding properties (Dupraz et al., 2009) and antibiotics led to a qualitative weakening of biofilms. Thus, symbiolite formation is based on two typical components of microbially induced calcification, (i) the alkalinity engine, in this case photosynthesis, and (ii) bacteria, which support calcification most likely by producing, degrading and modifying EPS components (Dupraz et al., 2009; Decho, 2010; Arp et al., 2012). And yet, symbiolite formation is to our knowledge the only microbial calcifying process that was discovered *in vitro*, i.e., without knowledge of a natural analog.

The ensuing question of whether *Symbiodinium*-induced PCP could in principle also occur in nature, was the primary motivation for the present study. Calcifying microbial communities in nature are subject to dynamic changes in photosynthesis and respiration and thus an ever-shifting balance between precipitation and dissolution of minerals (Visscher and Stolz, 2005; Gallagher et al., 2012; Shiraishi, 2012). In closed systems such as batch cultures, these dynamics can be amplified, creating conditions the cultured organisms would not experience in nature (Brewer and Goldman, 1976; Wanner and Egli, 1990). Consequently, symbiolite formation cannot simply be assumed to occur in natural environments. Another point to consider when asking the question of whether symbiolite formation could occur naturally concerns the critical influence of EPS properties on microbial calcification processes (Dupraz et al., 2009; Decho, 2010; Arp et al., 2012). EPS functional groups are typically not distributed homogeneously but are organized in microdomains on the nm to μm scale, giving biofilm matrices a microspatial structure that strongly influences their properties (e.g., Kawaguchi and Decho, 2002; Lawrence et al., 2007). These properties include both calcification-inhibiting, and -enhancing microdomains, but which of these domains dominate is in part determined by how the domains are structured on a microspatial scale (Dupraz et al., 2009; Decho, 2010) and how they are changed during dynamic processes of EPS production, degradation and modification (Arp et al., 2012). Resistance of the structural component of EPS to hydromechanical shear stress varies widely in naturally occurring biofilms (reviewed in Flemming and Wingender, 2010; Gerbersdorf and Wieprecht, 2015). For example, biofilms in dynamic systems with high levels of hydrodynamic shear stress are generally more resistant to this type of disturbance than biofilms grown under stagnant conditions (e.g., Jaeger-Zuern and Gruberg, 2000). Thus, the fact that the *Symbiodinium* cultures studied by Frommlet et al. (2015a) were not aerated nor agitated because of the well-known sensitivity of dinoflagellates to hydromechanical shear stress (reviewed in Peters and Marrasé, 2000), raises the questions of how naturally relevant flow conditions and disturbance of biofilm integrity would affect symbiolite formation.

Here, we studied symbiolite formation in batch culture, i.e., the *in vitro* condition under which symbiolites were first discovered in Frommlet et al. (2015a), and for the first time in natural seawater under laminar flow, in order to determine how algal growth dynamics, the physicochemical macroenvironment in cultures, and the microenvironment of *Symbiodinium*-bacterial biofilms influence the underlying calcification process. Together, these different lines of experimental studies provide insights into some of the processes that govern these novel calcifying communities and allow a first evaluation of how likely symbiolite formation is under natural conditions.

## Materials and methods

### *Symbiodinium* strains and culturing conditions

A total of 46 non-axenic *Symbiodinium* strains were used in this study (Table 1). Strains lacking published references, were identified to clade level based on partial ribosomal gene and internal transcribed spacer sequences, amplified according to Lajeunesse and Trench (2000) and Santos et al. (2001) (for details, see Supplementary Information). Stock cultures were routinely maintained in f/2 medium (Guillard, 1975) at 26°C and 130–150 μmol photons m^−2^ s^−1^ under a 12:12-h light:dark cycle and were subcultured monthly at a 1:40 ratio of culture to f/2 medium (for details, see Supplementary Information). Depending on the required volumes, experimental cultures were grown either in 24-well plates for suspended cell cultures (Sarstedt, Nürnbrecht, Germany), sealed with parafilm to prevent evaporation, or in 75 cm^2^ tissue culture flasks for suspended cell cultures with vented caps (Sarstedt, Nürnbrecht, Germany). Customized culturing flasks were used for microsensor measurements (see Supplementary Figure 1).

**Table 1:** *Symbiodinium* strains used in this study.

**Culture (NCMA no.)**	Species (^*^)	**Clade or ITS type (Ref.)**	**GenBank accession no**	**Host species**	**Geographic origin**
61 (2464)	*S. microadriaticum* Freudenthal 1962	A1 (1)		*Cassiopeia xamachana*	Florida
362 (2458)	*S. microadriaticum* Freudenthal 1962	A1 (1)		*Cassiopeia andromeda*	Gulf of Aqaba
370 (2467)	*S. microadriaticum* Freudenthal 1962	A1 (1)		*Stylophora pistillata*	Gulf of Aqaba
23		A2 (1)		*Bartholomea annulata*	Barbados
89		A2 (1)		*Gorgonia ventalina*	Bermuda
97		A2 (1)		*Gorgonia ventalina*	Puerto Rico
104		A2 (1)		*Heliopora* sp.	Enewetak
130		A2 (1)		*Meandrina meandrites*	Jamaica
185 (2461)	*S. pilosum* Trench and Blank 1987	A2 (1)		*Zoanthus sociatus*	Jamaica
PHMS TD1e		A3a (2)		*Tridacna* sp.	Philippines
292 (2465)		A3 (1)		*Tridacna maxima*	Palau
379 (2456)		A4 (1)		*Plexaura homomalla*	Bahamas
Culture X		A12 (2)		Unknown	Aquarium tank
80 (2469)	*S. necroappetens* LaJeunesse et al. 2015	A13 (1)		*Condylactis gigantea*	Jamaica
PTA1		A13 (2)		*Porites asteroides*	Caribbean, Florida
m. mirabilis		A14 (2)		*Madracis mirabilis*	Florida
FLAp1		A (3)		*Aiptasia* sp.	Florida Keys
Pk708		A (4)		*Plexaura kuna*	San Blas
AV32		A (this study)	MF616331	*Galaxea fascicularis*	Red Sea
99		A (this study)	MF616332	*Gorgonia ventalina*	Puerto Rico
105		A (this study)	MF616333	*Heliopora* sp.	Enewetak
107		A (this study)	MF616334	*Heliopora* sp.	Enewetak
108		A (this study)	MF616335	*Heliopora* sp.	Enewetak
154		A (this study)	MF616336	*Discoma sanctithomae*	Jamaica
2 (2460)	*S. minutum* LaJeunesse et al., 2012	B1 (1)		*Aiptasia pallida*	Florida
12 (2463)		B1 (1)		*Aiptasia tagetes*	Puerto Rico
13		B1 (1)		*Aiptasia tagetes*	Bermuda
64		B1 (1)		*Cassiopeia xamachana*	Jamaica
74		B1 (1)		*Cassiopeia xamachana*	Jamaica
Pk704		B1 (3)		*Plexaura kuna*	San Blas
Pk706		B1 (3)		*Plexaura kuna*	San Blas
146 (3450)	*S. pseudominutum* Parkinson et al., 2015	B1 (1)		*Oculina diffusa*	Bermuda
147 (2470)		B1 (1)		*Pseudoterogorgia bipinnata*	Jamaica
M. capitata		B1 (1)		*Montipora capitata*	Hawaii
351		B1 (1)		*Pocillopora damicornis*	Hawaii
Pk702		B2 (3)		*Plexaura kuna*	San Blas
141 (3320)	*S. psygmophilum* LaJeunesse et al., 2012	B2.1 (1)		*Oculina diffusa*	Bermuda
385 (2462)		B3 (2)		*Dichotomia* sp.	Caribbean
FLAp2		B (3)		*Aiptasia pallida*	Florida
203		C2 (1)		*Hippopus hippopus*	Palau
HHC1B		C2 (2)		*Hippopus hippopus*	Philippines
401		D (5)		Unknown	Unknown
Ap31		D (4)		Unknown anemone	Okinawa
383 (3420)	*S. voratum* Jeong et al., 2014	E1 (1)		*Anthopleura elegantissima*	California
135 (2468)	*S. kawagutii* Trench and Blank, 1987	F1 (2)		*Montipora verrucosa*	Hawaii
133 (2455)		F2 (1)		Meandrina meandrites	Jamaica

*NCMA, National Center for Marine Algae and Microbiota*.

* *Species names are provided for cultures that are deposited in a collection and are either identified to species level by the collection or could be matched with the holotype in the respective species description. (1) LaJeunesse, 2001; (2) LaJeunesse et al., 2005; (3) Santos et al., 2001; (4) Santos et al., 2002; (5) Thornhill and Lord, 2010*.

### Assessment of growth curves and onset of calcification in batch culture

*Symbiodinium* growth curves were determined for five strains under standard light and temperature conditions (see above). For this, triplicate cultures of each strain were inoculated with 25,000–50,000 cells mL^−1^ in f/2 medium and their growth was monitored until cultures reached stationary phase by performing manual cell counts of Lugol-preserved samples in a Nageotte counting chamber (for details, see Supplementary Information). To determine the onset of calcification under standard batch culture conditions, 45 calcifying *Symbiodinium* strains (Supplementary Table 1) were inoculated at a ratio of 1:40 of culture to f/2 medium and monitored microscopically for the appearance of symbiolites (for details, see Supplementary Information).

### Carbonate system, pH, and onset of calcification in undisturbed and disturbed batch culture

Duplicate batch cultures of four *Symbiodinium* strains were grown under standard batch culture conditions (see above) in 75 cm^2^ tissue culture flasks for suspended cell cultures with vented caps (Sarstedt). For each strain, one flask was kept undisturbed, while the other flask was disturbed several times a week to disrupt the microstructure of the developing biofilm (see results for specific days). Due to the experimental design, which included laborious microscopic screenings of the entire culture flask surface and manual titrations that were too time consuming to guarantee that DIC and related parameters would not change during the storage of experimental replicates (see below), the four *Symbiodinium* strains were studied without experimental replication and in two separate experiments. The first experiment was conducted with strains (clade/ITS type) AV32(A) and PK702(B1), the second with 74(B1) and 203(C2). During the second experiment, a sterile f/2 medium control was kept in the incubator, alongside cultures 74 and 203, as a control for abiotic changes in medium chemistry. Due to natural variability, the batch of seawater for the second experiment had slightly higher initial TA, pH, DIC, and Ω_arag_.

Disruption of developing biofilms was achieved by detaching cells and the biofilm from the culture flask's surfaces using a cell scraper (Orange Scientific, Braine-l'Alleud, Belgium), followed by repeated pipetting with a 10-mL micropipette to further disperse algal cells and break up the developing bacterial-algal biofilm into a fine suspension. Throughout culture growth, pH, total alkalinity (TA), total dissolved inorganic carbon (DIC), and the aragonite saturation state (Ω_arag_) were monitored in undisturbed and disturbed batch cultures, while microscopically screening for the appearance of symbiolites (for details, see Supplementary Information). Culture pH was measured using a WTW pH meter (Weilheim, Germany), followed by manual volumetric titrations to determine TA, using a Gran function plot method (Gran, 1950, 1952) in the online version of the Alkalinity Calculator software (U.S. Geological Survey; Rounds, 2012). Based on measured variables pH, TA, salinity and temperature (for details, see Supplementary Information), DIC and Ω_arag_ were calculated, using the Microsoft Excel macro CO2SYS (CO2Sys_v2.1, Pierrot et al., 2006).

### Assessment of symbiolite growth-limitation using image analysis

*Symbiodinium* strain 105 was grown in 12 × 2 mL of f/2 medium (Guillard, 1975) on a 24-well tissue culture plate for suspended cell cultures (Sarstedt) under standard batch culture conditions (see above). All cultures were screened daily by microscopy for calcification, i.e., the formation of symbiolites. As soon as calcification was evident in all cultures, symbiolite growth was monitored using image analysis according to Frommlet et al. (2015a). Once symbiolites had stopped growing in all cultures, all cultures were resupplied with vitamins and trace elements according to the f/2 medium recipe (control). In addition, three cultures each received as experiential treatment either (i) nitrogen (NaNO_3_) and phosphorus (NaH_2_PO_4_) according to the f/2 medium recipe, (ii) 10.87 mM calcium in form of CaCl_2_ (calcium concentration of modern seawater; Tyrrell and Zeebe, 2004), or (iii) a combination of the macronutrient and calcium treatment. To determine the effect of these treatments, monitoring of symbiolite growth by image analysis was continued and results were expressed relative to symbiolite size at the time of medium replenishment.

### Microsensor measurements in batch culture medium and in flowing natural seawater

*Symbiodinium* batch cultures for microsensor measurements were grown in f/2 medium under standard batch culture conditions (see above) using 75 cm^2^ tissue culture flasks for suspended cell cultures that were cut-open to provide physical access of microsensors (Sarstedt; for details, see Supplementary Information). Microsensor measurements in batch culture were performed directly in these modified flasks. For measurements in natural, flowing seawater, the growth medium was decanted, a self-built laminar flow chamber was introduced into the modified flasks, and the flasks were filled carefully with natural, sterile-filtered seawater without disturbing the biofilm (for details, see Supplementary Figure 1). Liquid ion-exchange (LIX) glass microsensors for pH and Ca^2+^ with a tip size of 10–25 μm were constructed as described previously (Ammann et al., 1987; de Beer et al., 1997, 2000). Electrochemical Clark type O_2_ microsensors were obtained from Unisense A/S (Aarhus, Denmark; Revsbech, 1989). Oxygen and pH dynamics were measured on the biofilm/symbiolite surface over several light-dark cycles. Depth profiles of O_2_, pH and Ca^2+^ were measured from the biofilm/symbiolite surface through the diffusive boundary layer into the overlying water column. Using dichlorophenyl dimethylurea (DCMU), a specific inhibitor of photosystem II (Bishop, 1958), we tested whether calcification could be suppressed by inhibiting photosynthesis. DCMU was dissolved in ethanol and added to seawater to a final concentration of 1 μM (Al-Horani et al., 2003). For such measurements, we increased the ambient flow to ensure effective mixing of DCMU in the experimental flow chamber. Calcium concentration profiles were measured before and 10 min after DCMU addition. For further technical details and for calculations of parameters, see Supplementary Information.

### Quantification of precipitated CaCO_3_ using LIX Ca^2+^ microsensors

For the determination of culture-specific, maximum amounts of precipitated CaCO_3_, 32 *Symbiodinium* strains, covering a wide range of phylotypes, were cultivated in 24-well tissue culture plates for suspended cell cultures (Sarstedt) under standard batch culture conditions (see above) for 56 days. Based on previous results (Frommlet et al., 2015a) and the data presented in the present study, this culturing period generally ensures that calcifying cultures reach a post-calcifying stage. To quantify the precipitated CaCO_3_, in these cultures, we established a protocol that involved the removal of growth medium, followed by the dissolution of the precipitated CaCO_3_ in HCl and the determination of the calcium concentration in solution using Ca^2+^ microsensors (for details, see Supplementary Figures 2, 3).

### Statistical analysis

One-way ANOVA was used to test for differences in the maximum amount of precipitated CaCO_3_ between different clades and to test for differences between calcification endpoints of symbiolite growth limitation experiments. When statistical differences were observed (*P* < 0.05), Tukey's HSD post-hoc comparisons were applied to determine which of the groups and experimental treatments were significantly different (see Supplementary Information).

## Results

### Calcification in relation to *Symbiodinium* growth, carbonate system dynamics, and culture disturbance

**Figure 1:**
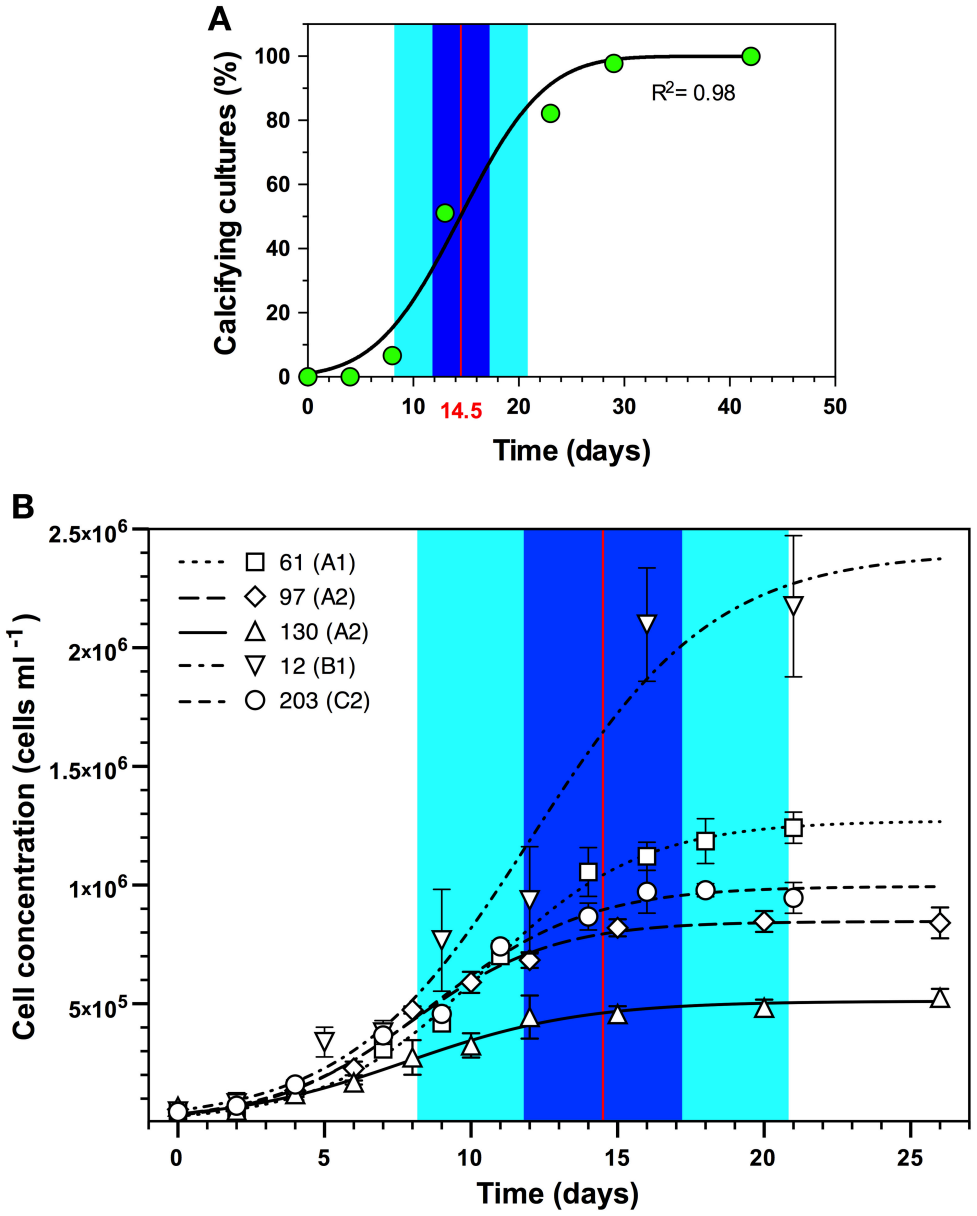
Onset of symbiolite formation in relation to *Symbiodinium* spp. growth dynamics. **(A)** *Symbiodinium* spp. cultures, inoculated 1:40 (~25,000–50,000 cells mL^−1^) in f/2 medium on average started to calcify after 14.5 days (red vertical line) with a 95% confidence interval from 11.8 to 17.2 days (Blue area) and a standard deviation of 6.3 days (light cyan area) (*n* = 45). **(B)** Projection of calcification dynamics in **(A)** onto growth curves of five of the 45 *Symbiodinium* strains (61, 97, 130, 12, and 203; ITS2-type designations in brackets) inoculated with 25,000–50,000 cells mL^−1^. Data are means ± s.d. (*n* = 3). Logistic functions were fitted to growth curves using the software Plot (version 1.997, http://plot1.micw.org).

Symbiolite formation in undisturbed *Symbiodinium* batch cultures started on average after 14.5 days (95% confidence interval [11.8–17.2]; *n* = 45) (Figure 1A; Supplementary Table 1). A comparison to growth curves of 5 of the 45 investigated *Symbiodinium* strains showed that the average onset of calcification corresponded to the late log- to early stationary growth phase (Figure 1B).

**Figure 2:**
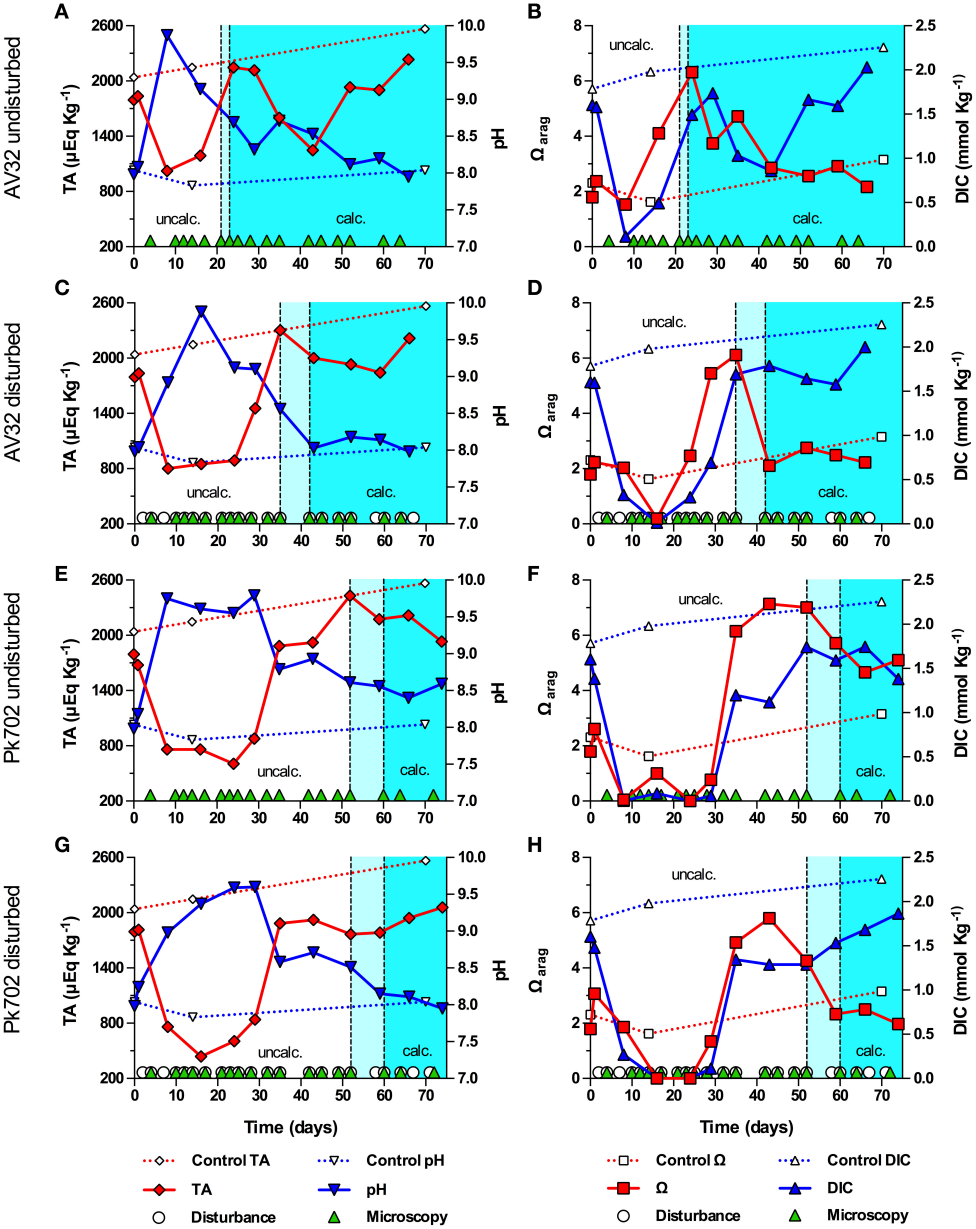
Onset of symbiolite formation in strains AV32 and Pk702 relative to carbonate system and culture disturbance. Total alkalinity (TA), pH, aragonite saturation state (Ω_arag_) and total dissolved inorganic carbon (DIC) in batch cultures of AV32 undisturbed **(A,B)**, AV32 disturbed **(C,D)**, Pk702 undisturbed **(E,F)**, and Pk702 disturbed **(G,H)**. A sterile f/2 medium control (dashed data lines) was kept under standard culturing conditions as described in section Material and Methods. Light cyan areas between vertical, dashed lines mark time windows during which cultures started to form symbiolites, i.e., transition from an un-calcified stage (uncalc., white background) to a calcified stage (calc., cyan background). Green triangles on the x-axis mark days on which cultures were screened microscopically for calcification (i.e., appearance and development of symbiolites). White circles on the x-axis in **(C,D,G,H)** mark days on which cultures were physically disturbed.

How the onset of calcification in undisturbed and disturbed batch cultures of *Symbiodinium* strains (clade/ITS2-type) AV32 (A) and Pk702 (B1) related to their pH and carbonate system dynamics is shown in Figure 2. In the undisturbed culture of strain AV32 (AV32_undist_), pH increased within the first 8 days from pH 7.98 to a maximum of pH 9.87, while total alkalinity (TA) decreased from 1795 μEq Kg^−1^ to a minimum of 1024 μEq Kg^−1^ (Figure 2A). By day 24 to 29, pH and TA had returned to values similar to those at the beginning of culture growth and those of a sterile f/2 medium control. Total DIC dynamics were similar to those of TA, decreasing from 1.61 mmol Kg^−1^ to a minimum of 0.11 mmol Kg^−1^ by day 8, before rising again to 1.74 mmol Kg^−1^ by day 29 (Figure 2B). Microscopic examinations showed that calcification started between day 21 and 23, when pH, TA, and DIC had already passed through their most extreme states and when the aragonite saturation state approached a maximum of Ω_arag_ = 6.32 on day 24 (Figures 2A,B). The calcifying phase was accompanied by a second, less pronounced increase of pH and Ω_arag_, which peaked on day 35 at pH 8.71 and 4.72, respectively, and a second decrease of TA to 1,249 μEq Kg^−1^ and of DIC to 0.86 mmol Kg^−1^ on day 43. Thereafter, all four parameters returned to values close to those of the medium control, indicating the end of the calcification phase.

In the disturbed culture of strain AV32 (AV32_dist_), pH increased to pH 9.88 on day 16 and TA decreased to ~800 μEq Kg^−1^ between day 8 and 24, before returning to values similar to the medium control around days 35–43 (Figure 2C). Changes in DIC mirrored the TA dynamics with a drop from 1.60 to 0.014 mmol Kg^−1^ on day 16 before returning to 1.69 mmol Kg^−1^ by day 35 (Figure 2D). After an initial drop 0.19 on day 16, Ω_arag_ increased to a maximum of 6.11 on day 35 and dropped again to ~2 by day 43 (Figure 2D). First symbiolites were evident in AV32_dist_ on day 42 (Figures 2C,D). Thus, calcification had commenced between day 35 and 42. Like in AV32_undist_, calcification coincided with a peak in Ω_arag_ but was accompanied by only a small decrease of TA and DIC until day 60, followed by a recovery toward the end of the 66-day experiment. Consistent with the small changes in carbonate chemistry, only few symbiolites formed in protected corners of the culturing flask that could not be disturbed as thoroughly as the rest of the culture.

Both undisturbed and disturbed cultures of Pk702 displayed pH and carbonate chemistry dynamics during the pre-calcifying phase that were similar to those in AV32. For instance, a temporary increase of pH and a temporary decrease of TA, DIC and Ω_arag_ was evident (Figures 2E–H). The time periods during which these initial changes, especially of pH, persisted were several days longer than in the AV32 cultures. Yet, as in both AV32 cultures, calcification only commenced when pH and the carbonate system had passed through their most extreme phases. When first symbiolites appeared, Ω_arag_ in Pk702_undist_ was still close to its maximum of 7.14 on day 43 and in Pk702_dist_ was still elevated at 4.26 (Figures 2E,F). The main difference to AV32_undist_ was that Pk702_undist_ calcified much later (between days 52 and 60) and produced only few and small symbiolites. The lower production of symbiolites, was reflected in the modest decline in TA and DIC. However, TA and DIC dynamics also indicated that calcification was still ongoing by the end of the experiment as both parameters had not yet started to recover from the calcification-associated decline. In Pk702_dist_, calcification commenced between days 52 and 60 but was minor and, as in AV32_dist_, restricted to corners of the culturing flask, causing no decrease in DIC and TA (Figures 2G,H).

**Figure 3:**
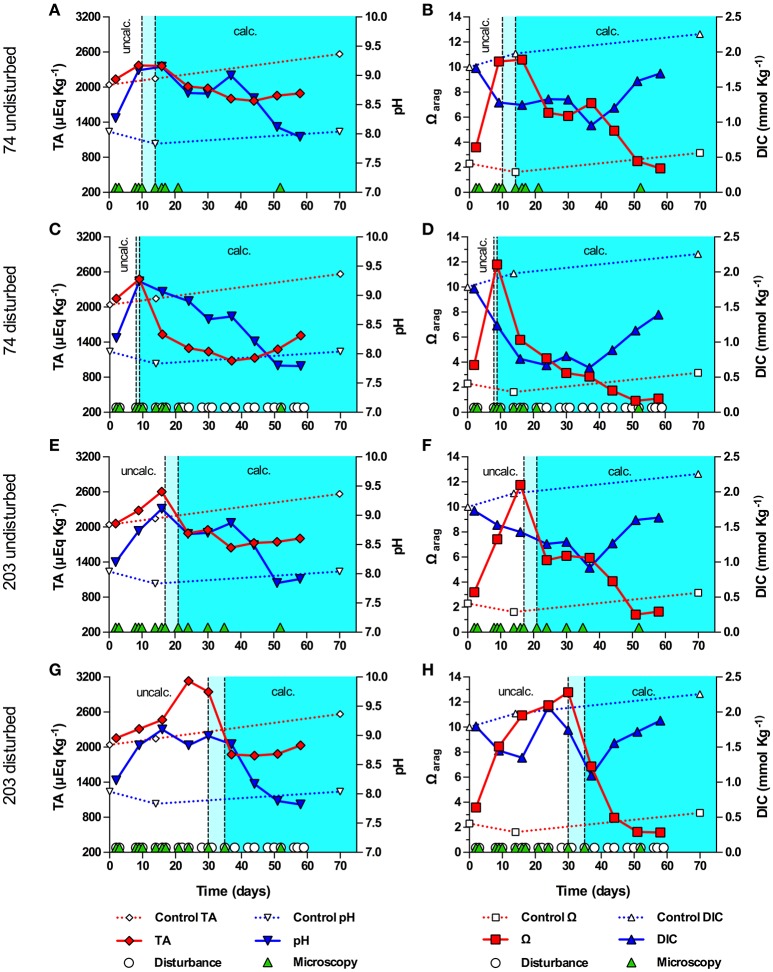
Onset of symbiolite formation in strains 74 and 203 relative to carbonate system and culture disturbance. Total alkalinity (TA), pH, aragonite saturation state (Ω_arag_) and total dissolved inorganic carbon (DIC) in batch cultures of 74 undisturbed **(A,B)**, 74 disturbed **(C,D)**, 203 undisturbed **(E,F)**, and 203 disturbed **(G,H)**. A sterile f/2 medium control (dashed data lines) was kept under standard culturing conditions. Light cyan areas between vertical, dashed lines mark time windows during which cultures started to form symbiolites, i.e., transition from a un-calcified stage (uncalc./white background area) to a calcified stage (calc., cyan background). Green triangles on the x-axis mark days on which cultures were visually screened for calcification on a microscope (i.e., appearance and development of symbiolites). White circles on the x-axis in **(C,D,G,H)** mark days on which cultures were physically disturbed.

A second experiment, conducted with strains (ITS type) 74 (B1) and 203 (C2), revealed that the pH in 74 and 203 were less strong than during the first experiment, with none of the cultures reaching a pH above 9.24 (Figure 3). In contrast to the first experiment, TA increased in all cultures during the pre-calcifying phase, decreased during the calcifying phase, and showed a recovery trend toward the end of the experiment (Figures 3A,C,E,G). All cultures showed a modest decline of DIC during the pre-calcifying phase with values remaining between 1 and 1.5 mmol Kg^−1^. With the exception of culture 203_dist_, DIC did not recover prior to calcification, kept decreasing during calcification, and recovered toward the end of the experiment (Figures 3B,D,F,H). In 203_dist_, DIC recovered during the pre-calcifying phase, then dropped associated with calcification and recovered toward the end of the experiment (Figure 3H); exhibiting dynamics comparable to those during the first experiment. Together, these differences resulted in a higher Ω_arag_, which peaked between 10.44 and 12.79 and, except for 203_dist_, were synchronized with the initial rise in pH and the onset of calcification. Culture 203_dist_ reached a peak in Ω_arag_ and began to calcify after the initial pH peak and DIC had recovered from its initial decrease (>9 days later than 203_undist_). Unlike in all other strains, disturbance promoted calcification in strain 74, as symbiolites appeared 1 and 7 days earlier in the disturbed culture than in the undisturbed culture.

Throughout experiments, the sterile f/2-medium control did not calcify and TA and DIC increased steadily from 2039 to 2566 μEq Kg^−1^ and from 1.79 to 2.26 mmol Kg^−1^, respectively, while pH and Ω_arag_ first decreased from pH 8.04 to 7.83 and from 2.31 to 1.62, respectively, and then rose to pH 8.04 and 3.15.

### Total CaCO_3_ precipitation in batch culture

**Figure 4:**
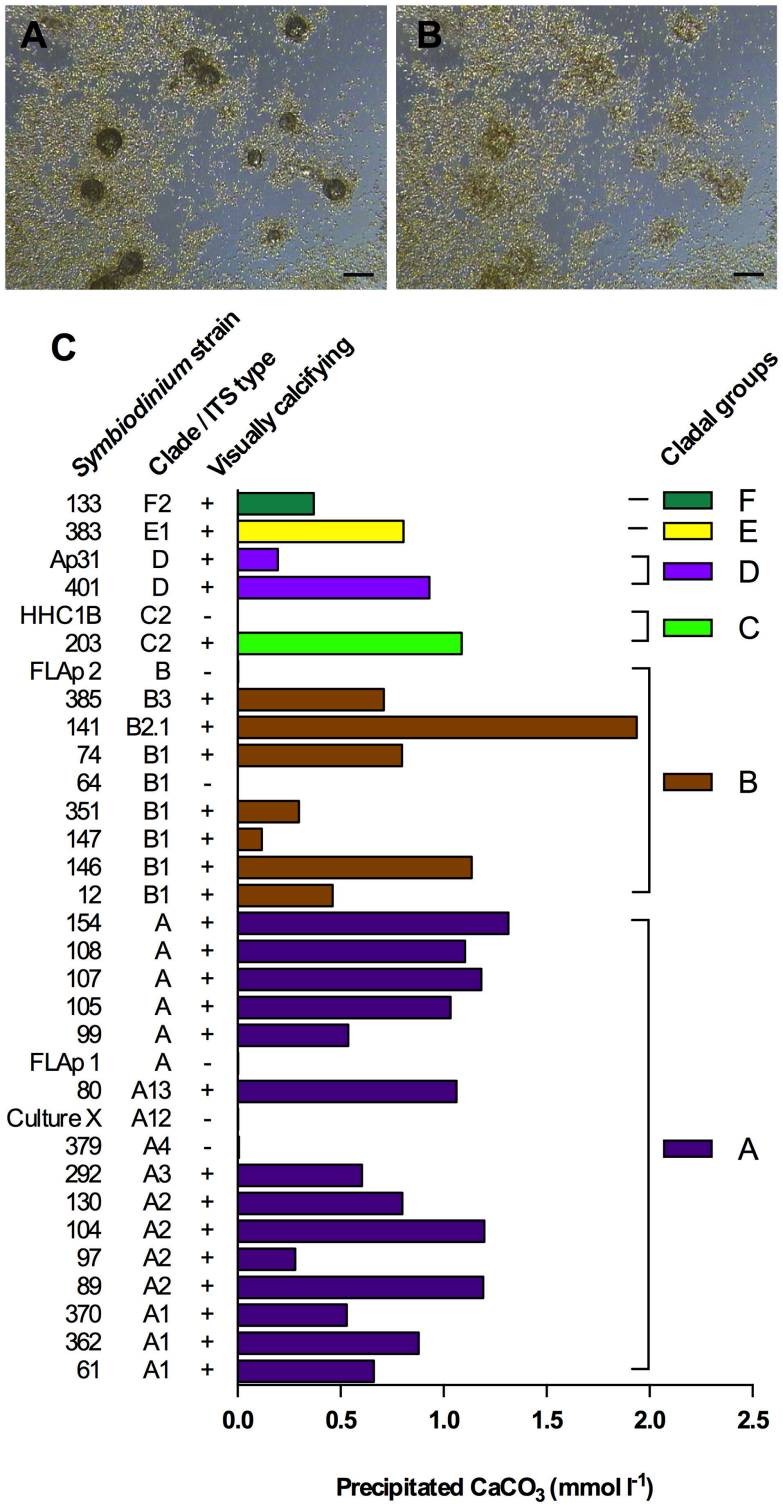
*Symbiodinium*-driven CaCO_3_ precipitation in batch culture. **(A,B)** Dissolution of symbiolites in a *S. minutum* culture (strain 12) using HCl (25 mM), to prepare the culture for Ca measurements. **(A)** 9 min after acidification, symbiolites are partially digested. **(B)** Same spot as in **(A)** after 35 min. Symbiolites are fully digested. Scale bar = 100 μm. **(C)** Final amounts of precipitated CaCO_3_ in 26 calcifying and 6 non-calcifying *Symbiodinium* strains after 56 days of batch culture growth. On day 56 of batch culture growth, prior to acid digestions, cultures were screened for symbiolite formation by microscopy (visually calcifying; ±).

Using a novel Ca^2+^ microsensor-based approach (Supplementary Figures 2, 3), we measured the total amount of precipitated CaCO_3_ in 26 visually calcifying and 6 visually non-calcifying *Symbiodinium* strains, representing 6 different clades and 12 different ITS2-types (Figures 4A–C). Excluding the non-calcifying cultures, total precipitated CaCO_3_ ranged from 0.12–1.94 mmol L^−1^ and averaged at 0.82 ± 0.42 mmol L^−1^ (mean ± *sd*). Amounts of precipitated CaCO_3_ in cladal groups with at least two strains (clades A, B, C and D) were not significantly different (ANOVA, *F* = 0.5460, *P* = 0.6663).

### Symbiolite growth limitation in batch culture

**Figure 5:**
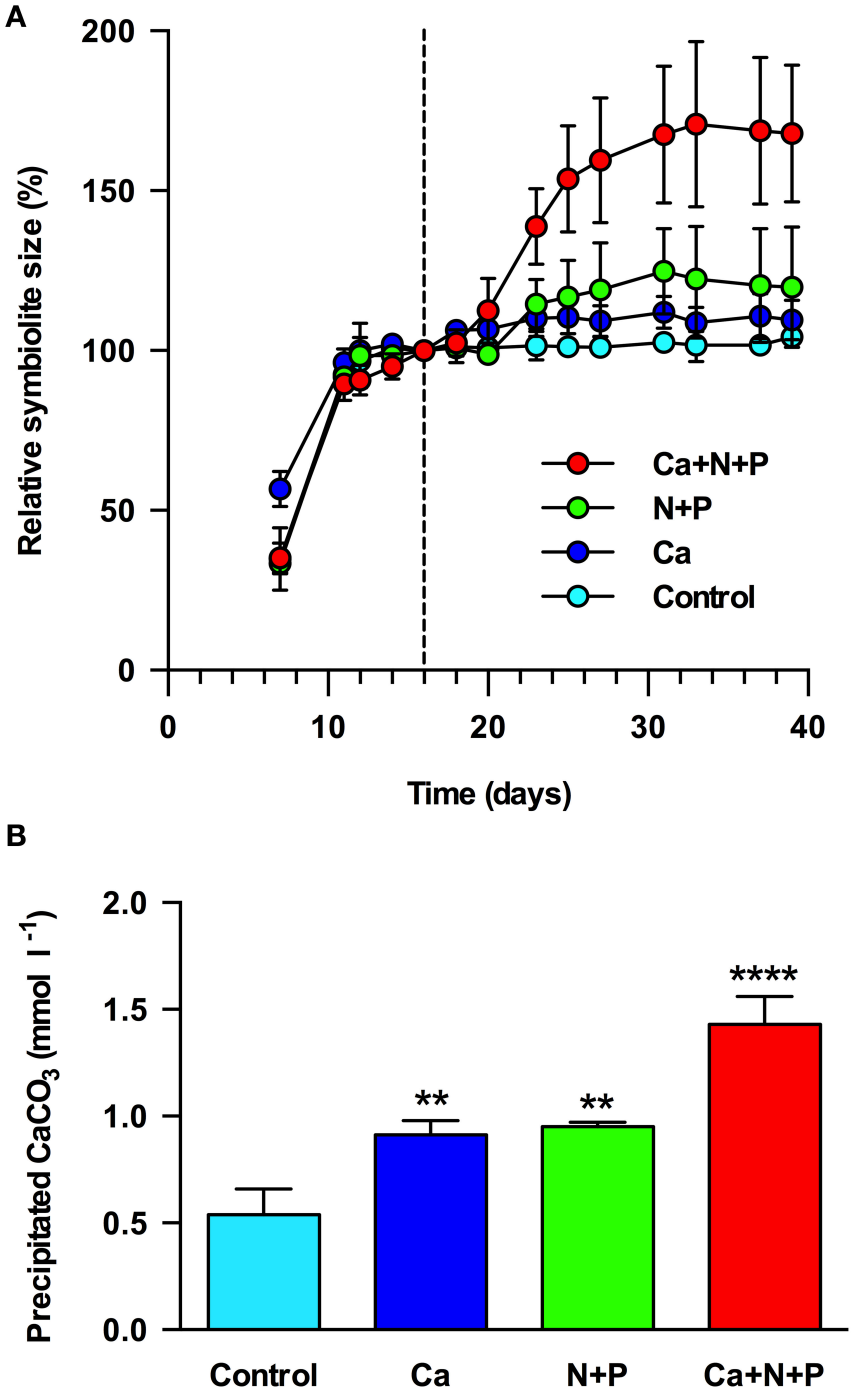
Limitation of symbiolite growth in batch culture. **(A)** Relative symbiolite size in batch cultures of *Symbiodinium* strain 105 before and after the medium was replenished on day 16 (marked by black, dashed line). Medium was replenished only with f/2 vitamins and trace elements (control) or in addition with calcium (Ca), nitrogen and phosphorus (N+P) or a combination of calcium, nitrogen and phosphorus (Ca+N+P). **(B)** Final amounts of precipitated CaCO_3_ at the end of the experiment (day 39) shown in **(A)**, measured by LIX Ca^2+^ microsensor. Asterisks indicate results from ANOVA where amounts of precipitated CaCO_3_ in treatments were significantly different from controls at ^**^P < 0.01, ^****^P < 0.001. Data are expressed as mean ± *sd*. (*n* = 3).

The replenishment of post-calcifying cultures with vitamins and trace elements did not stimulate additional symbiolite growth (Figures 5). Calcium caused a rapid but moderate increase in symbiolite size, evident 2 days after medium supplementation and leading to an increase of symbiolites by ~10%. Macronutrients led to an increase in symbiolite size by ~20%, which was evident after 7 days. The combined supplementation of calcium and macronutrients increased symbiolite size by ~70%. At the end of the experiment, quantitative measurements of calcification showed that the imaging-based approach had provided conservative estimates of the effects and that all treatments significantly increased growth relative to the control (Figure 5B; ANOVA; *P* = 0.001).

### Biofilm microenvironment in batch culture vs. flowing natural seawater for different irradiance regimes

**Figure 6:**
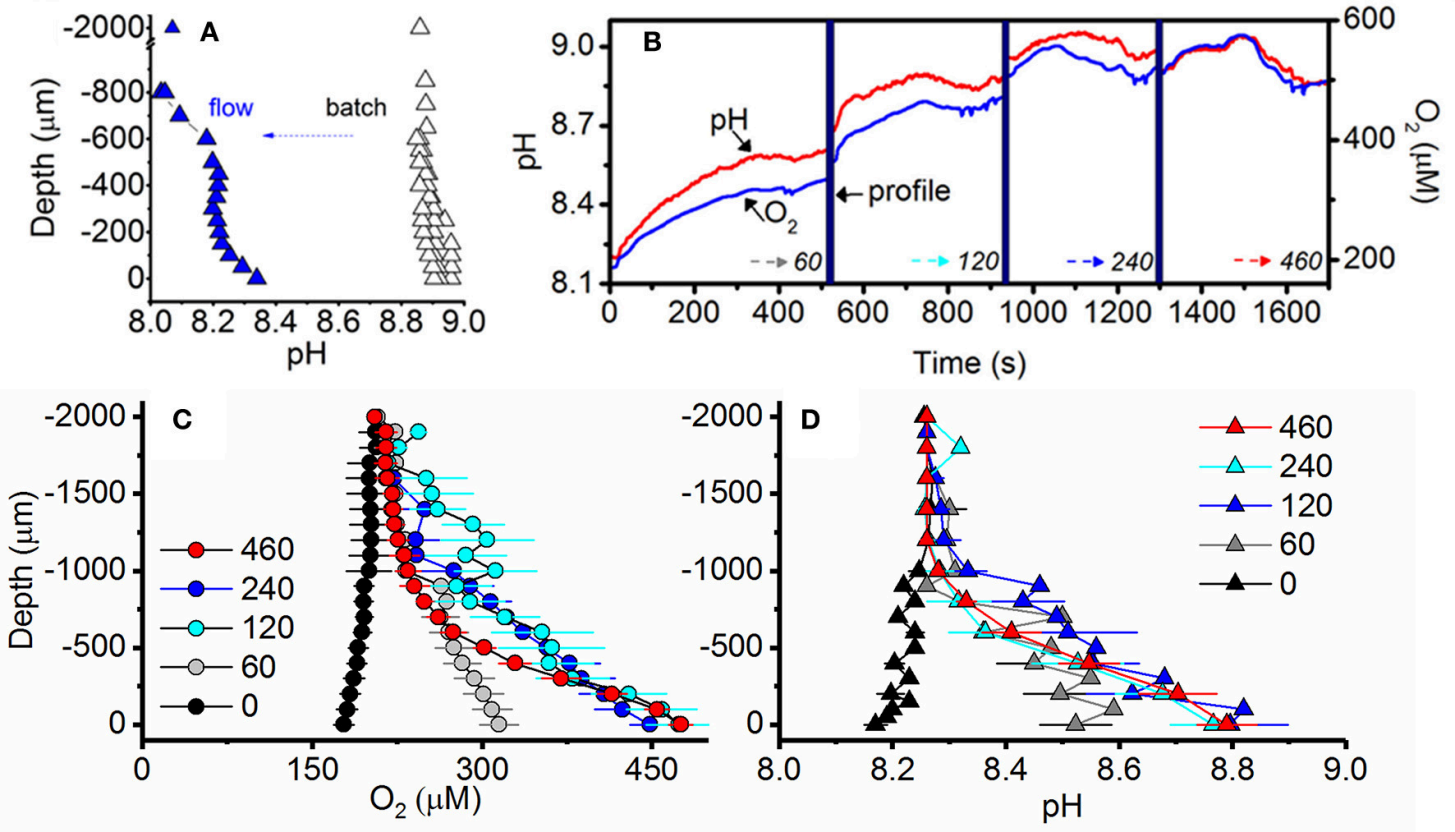
Irradiance and flow dependent changes of the O_2_ and pH microenvironment of symbiolites. **(A)** Comparison of pH under quasi-stagnant batch culture conditions (black) and under flow conditions (blue) measured consecutively in the same culture of strain AV32. Measurements were performed at an incident photon irradiance of 120 μmol photons m^−2^ s^−1^ under an ambient flow velocity of 0.5 cm s^−1^ (flow, *n* = 1) and under batch (quasi-stagnant) conditions (*n* = 3). **(B)** Example of simultaneous measurements of pH (red line) and O_2_ (blue line) dynamics at the surface of two adjacent calcifying symbiolites (separated by 200–700 μm distance) in a culture of AV32. Surface dynamics were measured in darkness (time = 0) and as a function of increasing photon irradiance (60, 120, 240, and 460 μmol photons m^−2^ s^−1^; indicated by dotted arrows). Microsensor depth profiles (time period shown as thick vertical blue line) were performed before irradiance was increased to a subsequent higher value. **(C)** O_2_ concentration and **(D)** pH profiles measured from the symbiolite surface (depth = 0 μm) into the overlying water column (negative values). Measurements were performed at increasing levels of incident downwelling photon irradiance (numbers denote μmol photons m^−2^ s^−1^). Data points with error bars represent means ± s.e. (*n* = 4–8 symbiolites).

pH microprofiles in a 10-day old, pre-calcifying batch culture (i.e., no flow and batch medium) of strain AV32 displayed an elevated medium pH of 8.8, typical for log phase batch cultures (compare to Figure 2) and a further rise of pH toward the biofilm microenvironment (pH = 8.9). Replacing the culture medium with a flow of natural seawater (0.5 cm s^−1^) reduced the pH in the bulk phase of the flow system to a regular seawater pH of ~8.1, while local photosynthesis within the biofilm microenvironment of this pre-calcifying culture elevated pH to ~8.4 (Figure 6A).

The O_2_ and pH microenvironment of actively calcifying biofilms of strain AV32 responded rapidly (within < 1 s) to changes in incident irradiance (Figures 6B–D). Under an ambient flow velocity of 0.5 cm s^−1^, the biofilm O_2_ concentration dropped to ~85% air saturation in darkness (178 ± 9.0 μmol O_2_ L^−1^; mean ± s.e.) and reached values of ~230% air saturation (475 ± 10.5 μmol O_2_ L^−1^; mean ± s.e.) at PAR levels >120 μmol photons m^−2^ s^−1^ (Figure 6C). These light-dependent changes in O_2_ concentration were accompanied by simultaneous changes in the pH microenvironment (Figure 6B). In darkness, the symbiolite surface pH was 0.09 pH units lower than that of ambient seawater (Figure 6D). Under PAR levels of >120 μmol photons m^−2^ s^−1^, pH-values at the symbiolite surface were 0.53 ± 0.07 pH units (mean ± s.e.) higher than the ambient seawater (Figure 6D). The thickness of the diffusive boundary layer (DBL) for both O_2_ and pH was ~1 mm at a flow velocity of 0.5 cm s^−1^ (Figure 6C–D).

### Photosynthetic activity and photosynthesis-dependent calcium precipitation

**Figure 7:**
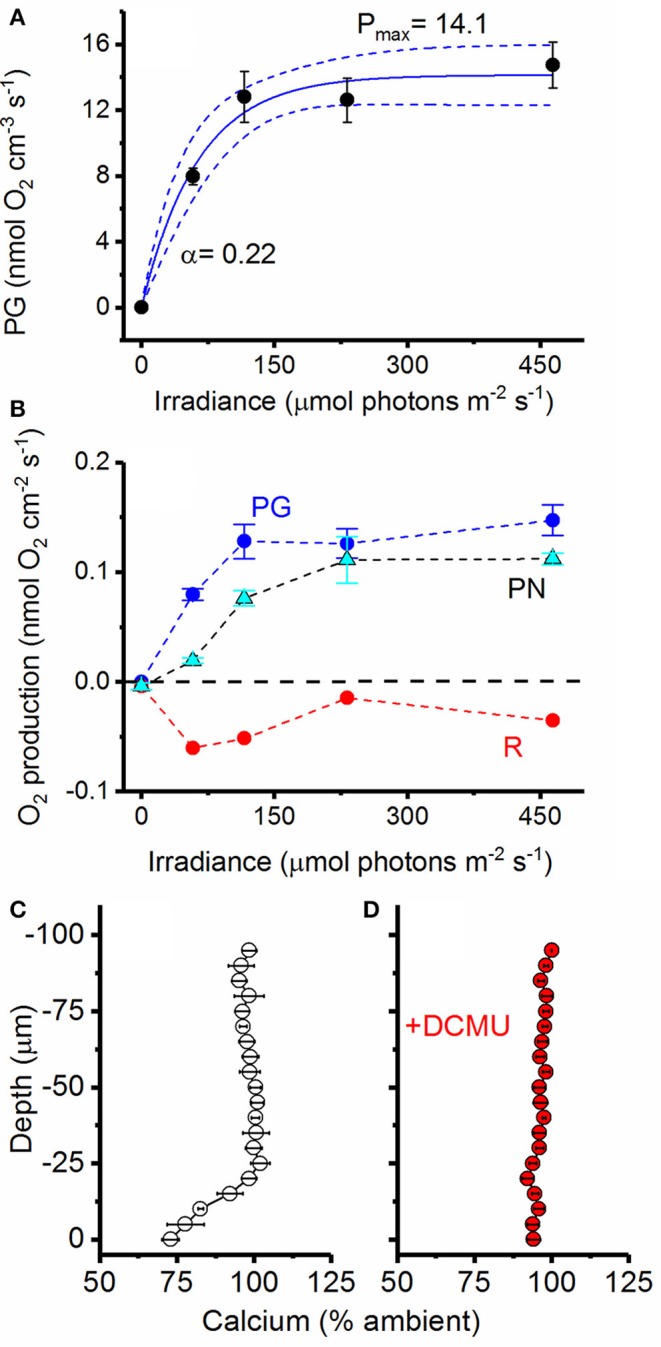
Photosynthetic activity and photosystem II dependent calcium uptake**. (A)** Volumetric gross photosynthetic rates (PG; black circles) measured at the surface of symbiolites (symbols with error bars represent means ± s.e.; *n* = 4). Data was fit to an exponential function (Webb et al., 1974, see section Materials and Methods) and the best fit (blue line, *R*^2^ = 0.74) and 95% confidence intervals (dotted blue lines) are shown. **(B)** Areal rates of gross photosynthesis (P_G_; blue), net photosynthesis (P_N_; cyan) and Respiration (R; red) (Symbols with error bars represent means ± s.e.; *n* = 4). The dotted black line marks the border between a net photosynthetic and net respiring system. **(C)** Active Ca^2+^ uptake measured at the surface of symbiolites with Ca^2+^ liquid-ion-exchange membrane microsensors under a photon irradiance of 460 μmol photons m^−2^ s^−1^. **(D)** The addition of DCMU (dichlorophenyl dimethyl urea), a strong inhibitor of photosystem II, led to the inhibition of calcium uptake (symbols with error bars represent means ± s.e.; *n* = 3).

Calcifying cultures were actively photosynthesizing (Figures 7A–D). Volumetric gross photosynthetic rates measured at the symbiolite surface under increasing levels of incident irradiance reached a maximum photosynthesis rate (P_max_) of 14.1 nmol O_2_ cm^−3^ s^−1^ with a light use efficiency factor α = 0.22 and an onset of photosynthesis saturation, E_k_ at ~75 μmol photons m^−2^ s^−1^ (Figure 7A). Net photosynthesis (P_N_) amounted to 22% and 54% of total gross photosynthesis (P_G_) at PAR levels of 60 and 120 μmol photons m^−2^ s^−1^, respectively (Figure 7B). Thus, calcifying biofilms respired about 78% and 46% of P_G_ at low to moderate light levels. At higher photon irradiance (>120 μmol photons m^−2^ s^−1^), light respiration decreased in proportion to P_N_, and P_N_ was about 80–85% of P_G_ (Figure 7B). Calcium microsensor measurements at the surface of symbiolites revealed active calcium precipitation in light (Figure 7C), which was inhibited ~5 min after the addition of the photosystem II inhibitor DCMU (Figure 7D).

### Microenvironments in calcifying vs. post-calcifying biofilms

**Figure 8:**
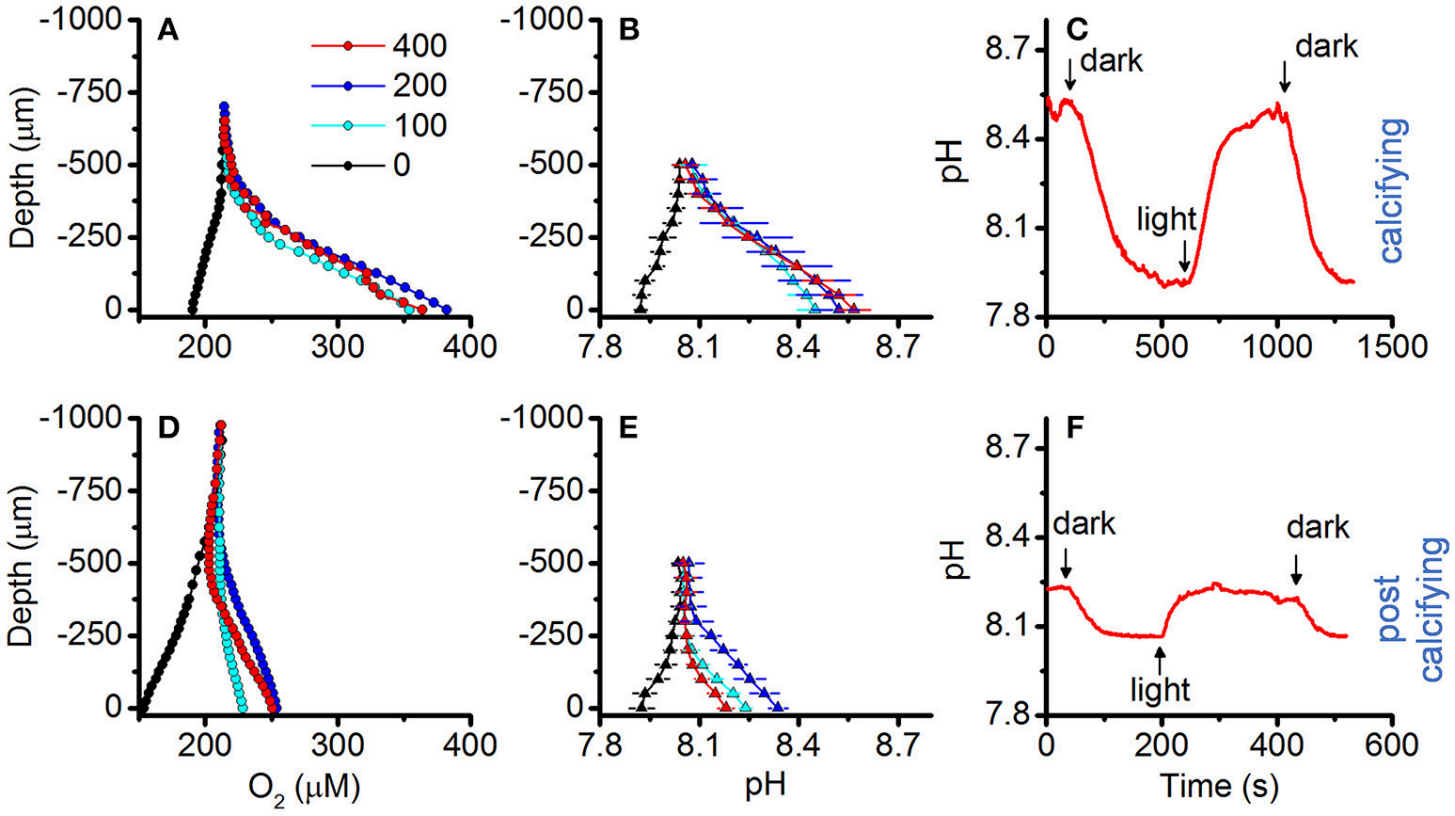
Effect of symbiolite age on O_2_ and pH microenvironmental dynamics. O_2_ and pH microsensor measurements were performed on actively calcifying symbiolites (days = 19; upper panel, **(A–C)** and post-calcifying symbiolites (days = 37, lower panel, **(D–F)** of *Symbiodinium* culture Pk702. **(A,D)** O_2_ and **(B,E)** pH microsensor measurements performed from the symbiolite surface (depth = 0 μm) into the overlying water column (negative values). Measurements were performed at increasing levels of incident downwelling irradiance (colors, in μmol photons m^−2^ s^−1^), Data points with error bars represent means ± s.e. (*n* = 2). **(C,F)** Example data of repeated light (100 μmol photons m^−2^ s^−1^) to dark cycles.

O_2_ and pH dynamics in the biofilm microenvironment of actively calcifying and post-calcifying cultures of strain Pk702 are shown in Figure 8. Actively calcifying cultures reached up to 190% air saturation (390 μmol O_2_ L^−1^), while post-calcifying cultures showed only moderate changes in O_2_, reaching maximal values of 120% air saturation (250 μmol O_2_ L^−1^; compare Figures 8A,D). Likewise, the biofilm pH of calcifying cultures ranged from pH 7.92 to 8.57 in darkness and light, respectively (Figure 8B), while pH in post-calcifying cultures ranged from pH 7.93 in darkness to pH 8.34 at 200 μmol photons m^−2^ s^−1^ and then decreased at a photon irradiance of 400 μmol photons m^−2^ s^−1^ to lower values than observed under 100 μmol photons m^−2^ s^−1^ (Figure 8E). This apparent inhibition effect was not pronounced for pH measurements performed on young cultures (Figure 8B). Repeated light-dark cycles showed maximal pH changes of 0.61 pH units vs. 0.17 pH units for calcifying and post-calcifying cultures, respectively (Figure 8C,F).

## Discussion

### Symbiolite formation relative to batch culture growth phase

One objective of the present study was to establish in which growth phase batch cultures become calcifying. However, culture dispersion, which is required to attain accurate cell counts for growth curves, influences the onset of calcification (see disturbance experiments). Thus, studying the onset of calcification in a culture relative to this culture's growth dynamics is practically impossible based on cell counts. Future studies may address this issue by using non-intrusive methods, e.g., optical density- or chlorophyll fluorescence measurements to estimate culture growth. Here we resorted to (i) determine the average time for undisturbed cultures to become calcifying based on microscopic screenings of all 45 calcifying strains used in this study, and (ii) to separately acquire growth curves from a phylogenetically diverse subset of these strains. Comparing the average time for calcification to commence (14.5 days) with the separately obtained growth curves showed that calcification typically commenced when cultures reached late log- to early stationary phase (Figure 1). Directly comparable data from the literature do not exist because few other studies on calcifying microbial communities have worked with experimentally grown biofilms, and these studies reported only very broadly on the growth stage of cultures or biofilms when calcification commenced (Hartley et al., 1996; Brehm et al., 2006). Constitutively biomineralizing microalgae such as coccolithophores calcify at the highest rate during log-phase, when growth rates and metabolic activities are typically highest (de Bodt et al., 2008). Considering that PCP is induced by photosynthesis (Arp et al., 2001; Dupraz et al., 2009; Shiraishi, 2012), one may thus expect that *Symbiodinium*-induced calcification would also commence during log-phase and not only toward stationary phase. However, as symbiolite formation is a bacterial-algal calcification process, the activity of the phototroph is but one of several factors driving calcification. Other important factors are the growth, the relative composition and the physiological activity of bacterial communities, as well as their production, modification, and degradation of EPS, and the structural development of the bacterial-algal biofilm (Dupraz et al., 2009; Decho, 2010; Arp et al., 2012). As shown by Frommlet et al. (2015a), bacteria are essential for symbiolite formation and are thought to be the main EPS producers. Because bacterial growth depends on photosynthates produced by *Symbiodinium*, a certain time lag between algal growth and the development of the bacterial community and their production and modification of EPS is likely, which could explain the late onset of calcification. Regarding the development of the bacterial-algal biofilm, it is also important to point out that under nutrient limitation and associated with the resulting reduction in cell division rates (Gunnersen et al., 1988; Rodríguez-Román and Iglesias-Prieto, 2005), an increasing number of *Symbiodinium* cells remain in the non-motile, coccoid stage during the photoperiod (Fitt and Trench, 1983). This change from motile zoospores to non-motile coccoid cells could be a key event in the development of the bacterial-algal biofilm and a decisive factor in initiating calcification, as it represents a major relocation of photosynthetic activity from the planktonic phase in the bulk medium into the surface-associated biofilm microenvironment. Under nutrient limitation, i.e., in a state of unbalanced growth, *Symbiodinium* may also excrete larger amounts of photosynthates (Davy et al., 2012), fueling bacterial growth and EPS production more efficiently during late stages of growth. Precisely how the bacterial communities and their metabolic activity develop in relation to *Symbiodinium* growth and how specific bacterial-algal interactions influence symbiolite formation are the focus of ongoing work.

### Symbiolite formation relative to the physicochemical conditions in batch culture

In general, batch cultures are not representative of the natural environment of a cultivated organism, as nutrients are initially available at unnaturally high concentrations, followed by their near-complete removal during growth. This leads to dense populations that strongly influence their physicochemical environment before they enter starvation (Wanner and Egli, 1990). Calcification-relevant factors, such as pH and the carbonate system are particularly affected by respiration and oxygenic photosynthesis (e.g., Brewer and Goldman, 1976). The two main objectives of this part of the study were therefore (i) to assess how culture growth affects the physicochemical conditions in batch culture and how these macroenvironmental conditions in the bulk medium relate to the initiation of symbiolite formation, and (ii), based on these data, to evaluate if symbiolite formation could also occur under natural conditions.

In strains AV32 and Pk702, maxima in pH and minima in DIC marked the time of highest photosynthetic activity, yet these cultures only became calcifying when both parameters had already relaxed substantially (Figure 2). The pH and carbonate system dynamics of these cultures thus confirmed that calcification typically sets in during late stages of growth, after cultures exerted their strongest influence on calcification-relevant parameters. To understand why the highest photosynthetic activity did not induce PCP, it is important to consider that photosynthesis-driven changes in pH and DIC have opposing effects on TA and Ω. More specifically, a photosynthesis-driven rise in pH shifts the DIC system toward ${\text{CO}}_{3}^{2-}$ and increases TA and Ω, while the accompanying CO_2_ assimilation decreases DIC and lowers TA and Ω (McConnaughey and Whelan, 1997; Riding, 2006; Shiraishi, 2012). It is this ambivalent effect of photosynthesis on Ω that explains why the strong rise of pH in AV32 and Pk702, indicative of logarithmic growth, did not lead to a high Ω_arag_ and calcification. Essentially, AV32 and Pk702 had depleted DIC to such an extent that even the strong rise in pH could not cause a high Ω_arag_. Only when pH-values indicated that cultures had passed through late log-phase, i.e., when bacterial respiration and/or diffusion of CO_2_ from the atmosphere into the medium caught up with CO_2_-assimilation, did DIC and TA recover sufficiently for Ω_arag_ to rise and for calcification to commence.

In experiments with strains 74 and 203, the drop in DIC was far less pronounced, TA remained high, pH did not become as alkaline, and Ω_arag_ reached higher values compared to AV32 and Pk702 and co-varied with the pH dynamics. Ultimately, these differences caused calcification to start earlier and at a time when, based on pH and carbonate system dynamics, cultures were still in log-phase. Independent of what caused these differences (see Supplementary Information for an extended discussion), these experiments showed that, given a large enough DIC pool, peaks in photosynthetic activity could induce calcification in batch cultures. Considering both cases: (i) onset of calcification not coinciding with highest photosynthetic activity and pronounced DIC depletion, combined with high maximum pH, and (ii) calcification coinciding with highest photosynthetic activity but under less extreme DIC and pH conditions, it can be concluded that *Symbiodinium*-driven calcification in batch culture is not the result of the most extreme conditions and that symbiolite formation is not merely an artifact of batch culture conditions.

### Effects of disturbance on physicochemical conditions in batch culture and symbiolite formation

Repeated physical disturbance influenced the onset of calcification in all four studied strains (Figures 2, 3). Considering that the properties of EPS and the biofilm matrix have important functional roles in bacterial-algal calcification and that these properties depend strongly on the microspatial structure of biofilms (Riding, 2000; Kawaguchi and Decho, 2002; Dupraz et al., 2009; Decho, 2010), it is likely that the inhibitory effect of biofilm disturbance on calcification in cultures AV32, Pk702 and 203 was caused by a disruption of the microspatial structure of the biofilm. However, in culture 74 disturbance actually promoted calcification, suggesting not only that this culture had a more resistant biofilm but that disturbance also had calcification promoting effects. Resistance of biofilms to hydromechanical shear stress is known to vary widely (reviewed in Flemming and Wingender, 2010; Gerbersdorf and Wieprecht, 2015), and thus it is not implausible that certain combinations of *Symbiodinium* strains and bacterial communities could form biofilms that are more resistant to physical disturbance than others and several other calcification-promoting and -inhibiting effects of disturbance are possible. A detailed assessment of these potential effects was beyond the scope of this study but see Supplementary Information for an extended discussion of possible effects. However, independent of the potentially complex effects, our results showed that physical biofilm disturbance generally had a negative effect on calcification but also that even severe physical biofilm disturbance by repeatedly dispersing the developing bacterial-algal biofilm into a fine suspension could not completely prevent calcification. Hence, the absence of significant hydromechanical shear stress, characteristic for batch cultures, does not appear to be an essential requirement for symbiolite formation, which increases the probability for this process to also occur in dynamic, natural environments.

### Quantitative assessment of calcification in batch culture

Previous data suggested that the genus *Symbiodinium* has broad potential to establish calcifying bacterial-algal communities and showed no correlation between *Symbiodinium* phylotypes and their potential to form symbiolites (Frommlet et al., 2015a). In this previous study, the calcification potential was assessed qualitatively by classifying strains as calcifying if they were at least once observed to form symbiolites. A Ca^2+^ microsensor-based approach (see Supplementary Information) now enabled the first quantitative assessment of *Symbiodinium*-induced microbial calcification (Figure 4). On average, calcifying cultures precipitated a total of 0.82 ± 0.42 mmoles CaCO_3_ L^−1^; equivalent to ~8% of the Ca^2+^ concentration in seawater (Tyrrell and Zeebe, 2004). Calcification varied widely between cultures but was not significantly correlated with *Symbiodinium* phylotypes, confirming on one hand a broad calcification potential of this genus but on the other hand indicating that calcification potential also depends on other factors, most likely functional differences in culture-associated bacterial communities. Five strains, previously reported as calcifiers, did not calcify during our experiments (e.g., FLAp 1 and HHC1B; compare to Frommlet et al., 2015a). This inconsistency in reaching a calcifying stage appears to be a feature of some of the weakly calcifying strains. Considering that microbial calcification is not tightly biologically controlled and that the process depends on several components, such as the physiological activity of heterotrophic and autotrophic partners and EPS dynamics, which in turn are all influenced by culture history prior to experimentation and slight variations in medium composition and light levels, such variations in calcification from one experiment to another are to be expected due to the complex nature of bacterial-algal calcification processes (e.g., Dupraz et al., 2009). It is therefore important to point out that the calcification amounts reported here only broadly define the calcification potential of *Symbiodinium*-bacterial associations.

Nevertheless, considering that (i) stationary phase *Symbiodinium* cultures typically reach ~10^6^ cells mL^−1^ (see Figure 1), (ii) the calcification phase lasts about 5–10 days (Frommlet et al., 2015a), and assuming cautiously that (iii) the entire algal population contributes to calcification, the average amount of total precipitated CaCO_3_ of 0.82 mmol L^−1^ corresponds to an average calcification rate of 0.082–0.164 pmol CaCO_3_ *Symbiodinium* cell^−1^ day^−1^, equivalent to 8.2–16.4 pg CaCO_3_ or 0.98–1.96 pg C *Symbiodinium* cell^−1^ day^−1^. Interestingly, these rates are not that dissimilar from those of strong calcifiers such as coccolithophores (1 pg C cell−1 d−1 in Trimborn et al., 2007; 7–22 pg C cell−1 d−1 in Zondervan et al., 2001). However, how important *Symbiodinium*-driven bacterial-algal calcification could be in terms of global calcification budgets, for example, compared to other microbial calcification processes (e.g., Heldal et al., 2012), can only be addressed if direct evidence for symbiolite formation is found in nature.

### Limitation of symbiolite growth in batch culture

Previous results showed that post-calcifying *Symbiodinium* cultures reacted to the replacement of their growth medium with additional symbiolite growth, suggesting that calcification was limited by a medium component (Frommlet et al., 2015a). In the present study, instead of replacing the medium entirely, we resupplied the original growth medium with specific medium components. This experimental design revealed that both calcium and macronutrient addition induced further symbiolite growth, and that their joint provision had a synergistic effect. However, at the time of these experiments we did not yet have the knowledge that batch cultures on average “only” remove ~8% of the total calcium from solution (Figure 4; Tyrrell and Zeebe, 2004). Consequently, replenishment of post-calcifying cultures with calcium, assuming that calcium had been substantially removed, overcompensated the actual calcium drawdown and must have resulted in ~20 mM calcium; a concentration the oceans have last seen tens of millions of years ago (Tyrrell and Zeebe, 2004). Therefore, the observed effect of the calcium treatment is interesting as it suggests increased symbiolite formation under calcium concentrations present in Earth's geological past. However, a near doubling of modern day calcium concentrations instantly increases the calcium ion activity {Ca^2+^}, which increases Ω and provides the potential for the observed fast re-initiation of calcification, without being a biologically meaningful indicator of whether calcification prior to the calcium addition was actually limited by calcium. In contrast, the calcification-promoting effect of macronutrients, explained by the necessity of macronutrients for algal photophysiology and the resulting effect on Ω (e.g., Shiraishi, 2012), must have been genuine because all presented data indicate that post-calcifying cultures were in stationary phase and thus medium replenishment did not overcompensate but only reinstated the original macronutrient status. Thus, symbiolite formation in batch culture is ultimately limited by the availability of macronutrients. The dynamics of nutrient availability in the nutrient poor surface waters of tropical coral reefs and the effects of these dynamics on the potential for symbiolite formation in nature will have to be addressed in future studies. However, the fact that coral reef sediments support high rates of organic matter mineralization and high primary productivity (Rasheed et al., 2004) and are known to calcify due to photosynthesis-driven microbial processes (Werner et al., 2008; Schoon et al., 2010; Cyronak and Eyre, 2016) show that in principle *Symbiodinium*-bacterial calcification should also be possible under natural nutrient conditions in reef habitats.

### Photosynthesis-induced calcification in unconditioned natural seawater

A comparison between pH profiles of a pre-calcifying culture of AV32 under batch culture conditions with the same culture once the medium had been replaced with flowing natural seawater, illustrates that the bulk medium (macroenvironment) of batch cultures strongly affects the biofilm microenvironment (Figure 6A). Yet, even in a young, pre-calcifying culture, photosynthesis in the biofilm alone, i.e., with the influence of the batch medium removed, was strong enough to cause a pronounced local increase in pH (Figure 6A). When reaching a calcifying stage, the same strain (AV32) displayed even more pronounced microenvironmental pH dynamics, reaching pH-values in the biofilm that were almost as high as those of the bulk medium during the pre-calcifying stage (compare Figures 5A,B,D). This increase of photosynthetic activity in the biofilm indicates that cultures, as they mature, develop a larger biofilm-associated algal population that eventually induces pH changes that are high enough to induce calcification. However, the fact that the highest pH measured in the calcifying culture was still lower than the bulk pH of the younger, pre-calcifying culture also confirms the idea that symbiolite formation is not simply a function of culture pH, and that other factors, related to the development of the bacterial-algal biofilm, are required to initiate calcification. Once these factors are in place, calcification does not require the extreme pH-values that are sometimes recorded in batch cultures (Figure 2).

Photosynthesis within the microenvironment of the bacterial-algal biofilm was very high, reaching a P_max_ of about 14 nmol O_2_ cm^−3^ s^−1^ (Figure 7A). Such high volumetric gross photosynthetic rates are similar to values obtained from corals (Kühl et al., 1995; Brodersen et al., 2014), and benthic foraminifera (Köhler-Rink and Kühl, 2000, 2001). Importantly, calcium profiles showed that these biofilms were actively calcifying without being influenced by macroenvironmental batch culture conditions. This proves that symbiolite formation can occur in unconditioned natural seawater and that moderate flow rates, indicative of coral reef lagoons (e.g., Jimenez et al., 2011), do not interfere with biofilm functionality in terms of its calcification potential. The fact that the photosynthesis inhibitor DCMU inhibited calcification provided further evidence that *Symbiodinium*-bacterial calcification is induced by the photosynthetic activity of *Symbiodinium* (Figure 7C,D). The thin Ca^2+^ boundary layer of ~25 μm in this experiment is explained by the fact that we increased the ambient flow to ensure effective mixing of DCMU in the flow chamber, leading to an erosion of the diffusive boundary layer. Finally, O_2_ and pH dynamics in actively calcifying *vs*. post-calcifying biofilms showed that with increasing age photosynthesis in *Symbiodinium*-bacterial biofilms decreases, while respiration increases (Figure 8), suggesting that arrest of calcification is related to the diminishing photophysiological activity in older cultures.

## Conclusions and perspectives

The experimental evidence presented here strengthens the notion that symbiolite formation is a typical bacterial-algal calcification process that is induced by photosynthesis of *Symbiodinium* spp. in the microenvironment of a bacterial-algal biofilm. Moreover, our data suggest that symbiolite formation is not merely an *in vitro* phenomenon but that this process should also occur under natural conditions. Whether *Symbiodinium* spp. actually drive bacterial-algal calcification and produce endolithic stages in natural habitats remains to be explored, but field studies that showed that (i) free-living *Symbiodinium* spp. occupy benthic reef habitat, especially reef sediments (e.g., Littman et al., 2008; Takabayashi et al., 2012), and (ii) that reef sediments calcify due to photosynthesis-driven calcification (Werner et al., 2008; Schoon et al., 2010; Cyronak and Eyre, 2016) provide first circumstantial evidence to support the novel concept of benthic *Symbiodinium* forming an endolithic life-stage by inducing the formation of microbialites. The prospect of a transient autoendolithic phase provides new perspectives on the biology and ecology of *Symbiodinium* and adds an entirely new ecological niche to be explored for *Symbiodinium* but also for dinoflagellates as a phylum. As outlined before by Frommlet et al. (2015a), an endolithic niche could be of important ecological relevance for free-living *Symbiodinium*, as it could act as a refuge from grazers and UV radiation, while still permitting photosynthesis (Friedmann, 1982; Shashar et al., 1997; Jeong et al., 2014). Finally, as *Symbiodinium* spp. are the first dinoflagellates known to drive a microbially induced mineralization process, symbiolite formation represents a valuable new model for the study of microbial calcification processes and the formation of microbialites. Current efforts in the study of symbiolites are directed at describing the bacterial communities and their role in biofilm formation and calcification, the assessment of potential functions of an endolithic stage for *Symbiodinium*, and the exploration of natural reef habitats for direct evidence of symbiolite formation and endolithic *Symbiodinium* populations.

## Author contributions

JF, DW, and JS designed and planned the study. JF, DW, MS, BG, and MM performed the research and analyzed the results. JF, DW, MK, and JS interpreted the data and wrote the manuscript. All authors approved the final version of the manuscript.

### Conflict of interest statement

The authors declare that the research was conducted in the absence of any commercial or financial relationships that could be construed as a potential conflict of interest.
