# Supplementary material for: Symbiodinium-Induced Formation of Microbialites: Mechanistic Insights From in Vitro Experiments and the Prospect of Its Occurrence in Nature
Source: Front Microbiol. 2018 May 17;9:998. doi: 10.3389/fmicb.2018.00998 (PMC5966549; doi:10.3389/fmicb.2018.00998)
Supplement: Supplementary file 1 [file Presentation_1.pdf]

Supplementary Material

Symbiodinium-induced formation of microbialites: Mechanistic insights from *in vitro* experiments and the prospect of its occurrence in nature

Jörg C. Frommlet[*1], Daniel Wangpraseurt[2,3], Maria L. Sousa[1,4], Bárbara Guimarães[1,5], Mariana Medeiros da Silva[6], Michael Kühl[2,7], João Serôdio[1]

*** Correspondence:** Jörg C Frommlet: jfrommlet@ua.pt

Contents

1. Supplementary Material and Methods

2. Method development for the quantification of precipitated $CaCO_3$ in batch culture

3. Extended discussion of variations in DIC limitation between experiments in batch cultures

4. Extended discussion of effect of culture disturbance on symbiolite formation

5. Supplementary References

6. Supplementary Figures 1-3

7. Supplementary Table 1

1. Supplementary Material and Methods

Symbiodinium strains and culturing conditions

Symbiodinium strains were kindly provided by Dr. Mark Warner (University of Delaware, Newark, DE, USA) and by Dr. Christine Ferrier-Pagès (Centre Scientifique de Monaco, Monaco) (Table 1). Strains lacking published references of their identity were identified to clade level based on full length internal transcribed spacer 1 (ITS1)-5.8S rDNA-ITS2 sequences or full length ITS2 sequences. Preparation of DNA for PCR amplification was based on a protocol originally developed for single cell PCRs of another dinoflagellate taxon (Frommlet and Iglesias-Rodriguez, 2008). Briefly, 10-25 µL of culture were pelleted for 5 min at 2000 rcf, the supernatant was removed, 6 acid-washed and sterile glass beads (0.7–1 mm; Sigma-Aldrich, St. Louis, MO, USA) and 10 µL of sterile Milli-Q water were added and the cells were physically broken up by vortexing at maximum speed for 1 min on a mini vortex mixer (VWR, Lutterworth, UK). As PCR template, 1 µL of this preparation was transferred to a 0.2 mL PCR tube. To amplify the entire ITS1-5.8S-ITS2 sequence, PCRs were carried out according to Santos *et al.* (2001). Full length ITS2 sequences were amplified according to LaJeunesse and Trench (2000). Amplicons were purified using the QIAquick PCR Purification Kit (Qiagen, Hilden, Germany). Sequencing was performed at STAB vida (Lisbon, Portugal) in forward and reverse direction, using the same primers that were used in initial PCRs.

Sequences were aligned using the software Bioedit, version 7.0.9.0 (Hall, 1999; http://www.mbio.ncsu.edu/bioedit/bioedit.html) and consensus sequences were searched against the GenBank database, using BLAST (National Center for Biotechnology Information, NCBI; https://blast.ncbi.nlm.nih.gov/Blast.cgi) to identify the closest phylogenetic relatives. The here reported six sequences have been deposited in the GenBank database under accession numbers MF616331–MF616336 (Table 1).

Symbiodinium cultures were routinely grown in f/2 medium, based on Atlantic seawater collected from the coast near Aveiro, Portugal without added silicate (Guillard, 1975). The salinity of each batch of seawater was measured using a V^{2}Refractometer (TMC, Chorleywood, UK) and was consistently at 36. Unless stated otherwise, f/2 medium was also used for experimentation. Cultures were grown at 26 °C and with a photosynthetically active radiation (PAR; 400-700 nm) of 130–150 µmol photons m^{-2} s^{-1} under a 12-h:12-h light:dark cycle. Stock cultures were subcultured monthly at a 1:40 ratio of culture to f/2 medium. Depending on the required volumes, experimental cultures were grown either in 24-well plates for suspended cell cultures (Sarstedt, Nürnbrecht, Germany), sealed with parafilm to prevent evaporation, or in 75 cm^2 tissue culture flasks for suspended cell cultures with vented caps (Sarstedt, Nürnbrecht, Germany). Cultures for microsensor measurements were grown in the same type of tissue culture flask but with an added opening to allow access of microsensors (Supplementary Figure 1A; see below for details).

Assessment of growth curves and onset of calcification in batch culture

A joint assessment of *Symbiodinium* growth dynamics and calcification was not possible, because culture dispersion to attain accurate cell counts itself influenced the onset of calcification (see Figures 2 and 3). Therefore, *Symbiodinium* growth curves were determined separately for five strains from four different ITS-phylotypes. For each strain, triplicate cultures were inoculated with 25,000–50,000 cells mL^{-1} in f/2 medium and cultivated under standard light and temperature conditions. Prior to sampling for cell counts, cells were detached from the culture vessel using a cell scraper (Orange Scientific, Braine-l'Alleud, Belgium), followed by repeated pipetting to disperse cells evenly. Samples were preserved in Lugol´s iodine at a ratio of 40:1. The Lugol´s iodine stock solution consisted of 10 g crystalline iodine, 20 g of potassium iodide, 200 mL H_2O and 20 mL of glacial acetic acid (Rodhe *et al.*, 1958) and was GF/F-filtered (Whatman, GE Healthcare, Piscataway, NJ, USA) prior to use. Cell concentrations were determined in triplicate counts using a Nageotte counting chamber (Paul Marienfeld GmbH & Co. KG, Marienfeld, Lauda-Königshofen, Germany) and a Leitz Laborlux S light microscope (Wetzlar, Germany). Fitting of logistic functions to cell concentrations over time was performed using the software Plot (version 1.997; http://plot1.micw.eu).

To determine the onset of calcification under batch culture conditions, 45 calcifying *Symbiodinium* strains (Supplementary Table 1) were inoculated at a ratio of 1:40 into 24-well plates and monitored for the appearance of symbiolites at seven time points by systematic microscopic screening using an inverted light microscope (Leica DM IL, Leica Microsystems, Wetzlar, Germany). The average time for the onset of calcification, a 95% confidence interval and the standard deviation were determined by fitting a cumulative Gaussian curve to the number of calcifying cultures over time, using Prism 6 for Mac OS X (GraphPad Software, Inc., San Diego, CA, USA).

Carbonate system, pH and onset of calcification in undisturbed and disturbed batch culture

Duplicate batch cultures of four *Symbiodinium* strains were grown under standard batch culture conditions in 75 cm^2 tissue culture flasks for suspended cell cultures with vented caps (Sarstedt, Nürnbrecht, Germany). For each strain, one flask was kept undisturbed, while the other flask was disturbed several times a week (see results for specific days) by detaching cells and biofilm from the culture flask´s surfaces using a cell scraper (Orange Scientific, Braine-l'Alleud, Belgium), followed by repeated pipetting with a 10-mL micropipette (Eppendorf, Hamburg, Germany) to further disperse algal cells and break up the developing bacterial-algal biofilm into a fine suspension. Throughout culture growth, pH, total alkalinity (TA), total dissolved inorganic carbon (DIC), and the aragonite saturation state (Ω_{arag}) were monitored in undisturbed and disturbed batch cultures, while microscopically screening for signs of calcification, i.e. the appearance of symbiolites. These screenings were performed systematically by inspecting the entire bottom of culture flasks using an inverted light microscope (Leica DM IL, Leica Microsystems, Wetzlar, Germany). All handling and sampling was performed 5-7 h into the light cycle to assure that cultures were consistently in the same photoperiodic stage and in the following order: Microscopic screening, sampling for chemical analyses and culture disturbance.

Total alkalinity was determined by manual volumetric titrations, which could be performed in 5 mL samples and thus kept culture volumes manageable. In comparison, automatic TA titrators typically require 50-100 mL, meaning that comparatively large cultures are required for repeated measurements. In our case, samples of 6 mL were drawn and gently filtered through sterile, 0.2 µm, cellulose acetate, syringe filters (Frilabo, Maia, Portugal). 5 mL of the cell-free filtrate were transferred to a 10-mL beaker and stirred gently with a magnetic stirrer. Using a WTW pH meter with a SenTix41 probe (WTW, Weilheim, Germany), the culture pH was measured, followed by manual volumetric titrations with 0.16 N sulfuric acid to values below the final endpoint (pH <3). Acid was added in steps of 0.5-10 µL with a micropipette (Eppendorf), ensuring that at least three data points were collected per pH unit. Titration data were analyzed in the online version of the Alkalinity Calculator software (U.S. Geological Survey; Rounds, 2012), using a Gran function plot method (Gran, 1950; 1952). DIC and Ω_{arag} were calculated, using the Microsoft Excel macro CO2SYS (CO2Sys_v2.1, Pierrot *et al.*, 2006). The aragonite saturation state is defined as $\Omega_{arag} = \{Ca^{2+}\} \cdot \{CO_3^{2-}\}/K_{sp}$, where $\{Ca^{2+}\}$ and $\{CO_3^{2-}\}$ denote the activities of each ion and K_{sp} denotes the solubility product of aragonite (Mucci, 1983). Measured input data were salinity (36), temperature (average temperature during manual titrations), and pH, and TA. Input data for total phosphorus was kept constant at an assumed average concentration of 17.66 µmol Kg^{-1}; equal to 50% of the concentration added to f/2 medium (Guillard, 1975), and total silicon was kept constant at 10 µmol Kg^{-1}, based on published values for the Portuguese coastal upwelling zone (Silva *et al.*, 2009). Further, pH was set to the NBS scale and the equilibrium constants K1 and K2 of carbonic acid were based on data in Hansson (1973) and Mehrbach *et al.* (1973) refitted by Dickson and Millero (1987).

Assessment of symbiolite growth limitation using image analysis

Symbiodinium strain 105 was inoculated at a ratio of 1:20 in a 24-well plate (12 x 2 mL cultures), grown under standard conditions as described above and checked daily for formation of symbiolites using a Leica DM IL inverted light microscope. As soon as calcification was evident in all cultures, symbiolite growth was monitored using image analysis. For this, one individual symbiolite in each culture was photographed repeatedly over the course of several days using a digital camera (Nikon D5100, Nikon, Tokyo, Japan). Manual measurements of symbiolite size, i.e., the area at the symbiolite base, were performed in triplicates, using the freehand tool of the image analysis software

ImageJ (U.S. National Institutes of Health). Once symbiolites had stopped growing in all cultures, all cultures were resupplied with vitamins and trace elements according to the f/2 medium recipe (control). Vitamins and trace elements were included as part of the control treatment because pilot experiments had shown that the addition of vitamins and trace elements did not lead to additional symbiolite growth (results not shown). As experimental treatments, 3 cultures received in addition 10.6 mM calcium in form of $CaCl_2$ to increase the unknown remaining calcium concentration by the concentration in modern seawater without affecting the DIC pool (Tyrell and Zeebe, 2004) (treatment 1). Three cultures received in addition nitrogen ($NaNO_3$) and phosphorus (NaH_2PO_4) according to the f/2 medium recipe (treatment 2), and 3 cultures received in addition a combination of calcium, nitrogen and phosphorus at the same concentrations (treatment 3). To determine which medium component limited calcification and thus symbiolite growth in batch culture, additional symbiolite growth induced by these treatments was assessed by image analysis as described above. Growth dynamics were expressed relative to symbiolite size at the time of medium replenishment (defined as 100%), as it marked the maximum size of symbiolites under standard batch culture conditions.

Microsensors, calibration, control and data acquisition

We used liquid ion-exchange (LIX) glass microelectrodes for pH and Ca^{2+} with a final tip size of 10-25 µm and constructed as described previously (Ammann *et al.*, 1987; de Beer *et al.*, 1997; de Beer *et al.*, 2000) as well as electrochemical Clark type O_2 microsensors (Unisense A/S Aarhus Denmark; Revsbech, 1989). To minimize noise and signal drift, LIX sensors were shielded against temperature and light fluctuations by wrapping the sensors in aluminium foil. Standard reference electrodes (Unisense A/S) were used to establish a reference potential against the pH and calcium microsensors. Both, reference electrode and potentiometric microelectrodes were connected to either a custom-built high impedance mV meter or, alternatively, to a commercial microsensor pH meter (Unisense A/S). Microsensors for pH were calibrated against standard pH buffers (pH = 4, 7 and 10; Fluka, Sigma-Aldrich, USA). The sensors showed a log-linear response to [H^+] with a signal change of 50-60 mV per pH unit. Calcium microsensors (10-20 µm tip size) were calibrated in NaCl solutions with 0, 0.5, 1, 10 and 20 mM $CaCl_2$. O_2 microsensors (10-50 µm tip size) were connected to a pA meter (PA2000, Unisense A/S) and were linearly calibrated from sensor signal readings in air-saturated water and anoxic water (flushed with N_2) at experimental temperature and salinity. Microsensors were mounted at a 45° angle relative to the vertical in two separate motorized micromanipulators (Pyro-Science GmbH, Aachen, Germany; L.O.T.-ORIEL, Märzhäuser GmbH, Wetzlar, Germany) that were fixed onto heavy-duty vibration-free metal stands. Data acquisition was done by interfacing the microsensor meters to a strip-chart recorder (BD25, Kipp&Zonen, Delft, The Netherlands) and via an A/D converter (Pyro-Science GmbH) to a PC running the microsensor positioning and data acquisition software Profix (Pyro-Science GmbH).

Culturing system and experimental setup for microsensor measurements

To provide physical access of microsensors to undisturbed biofilm, cultures were grown in 75 cm^2 tissue culture flasks (Sarstedt), from which the topside was cut out and replaced with a transparent removable acrylic lid (Supplementary Figure 1A). During cultivation, the lid was attached and sealed with parafilm to prevent contamination and water evaporation. To create laminar, unidirectional flow and a defined diffusive boundary layer (DBL) during measurements, we inserted a self-built laminar flow unit into the open tissue culture flasks (Supplementary Figure 1B-D) and pumped sterile-filtered (0.22 µm, Steritop, Merck Millipore KGaA, Darmstadt, Germany) natural seawater (salinity = 36)

into the chamber from a 1 L reservoir that was temperature-controlled (26°C) with a recirculating water bath (Frigiterm-10, Selecta, Barcelona, Spain) (Supplementary Figure 1E-G). Water flowed back into the reservoir by gravity through the neck of the culture flask, which was fitted with a short piece of rubber hose (Supplementary Figure 1G). Prior to their use, all culturing and laminar flow components were autoclaved or disinfected with 70% ethanol, and were additionally sterilized by exposure to UV-C radiation for 20 min (G20T10 Sankyo Denki, Japan) in a laminar flow sterile bench (BH-EN 2003 Faster, Italy). The assembled flow chamber was tested for turbulence by examining ink traces that were added to the flow using a micropipette. No turbulent flow was evident over the entire chamber. The flow rate during experimentation was ~0.5 cm s^{-1}, as determined by particle tracing. Such flow velocities can occur on coral reefs under low flow conditions such as in lagoons (Jimenez *et al.*, 2011). Microsensor measurements were performed with the assembled laminar flow chamber mounted on a Leica DM IL inverted light microscope (Supplementary Figure 1E), which allowed for the precise alignment of microsensors with individual symbiolites (Supplementary Figure 1H, I). Incident illumination was provided vertically from above at defined PAR levels (50, 116, 250 520 μmol photons m^{-2} s^{-1}) by a fiber-optic tungsten halogen lamp (460-F & KL-2500 LCD, Schott, Mainz, Germany). This temperature- and light-controlled setup was suitable to maintain cultures for extended periods of time outside the incubator.

Microsensor measurements in laminar flow chamber

To test for light-dependent changes in the O_2 and pH microenvironment of symbiolites, two microsensors were employed simultaneously. The microsensors were carefully positioned at the surface of symbiolites using the motorized micromanipulators. To avoid that the microsensors affected each other by altering the diffusion geometry, they were positioned at adjacent symbiolites of similar size that were not in direct but in close proximity to each other (within a few 100 μm) (Supplementary Figure 1H, I). Oxygen and pH dynamics were measured on the symbiolite surface over several light-dark cycles. Illumination/darkness was provided until steady state was reached, which usually took 5-10 minutes.

Depth profiles of O_2 and pH were measured from the symbiolite surface through the DBL into the overlying water column at defined PAR levels (60, 120, 240 460 μmol photons m^{-2} s^{-1}; see above). Diffusive fluxes of O_2 (nmol O_2 cm^{-2} s^{-1}), J, were calculated from steady state O_2 profiles using Fick's first law of diffusion:

$$J = -D\frac{\delta C(z)}{\delta z},$$

where D is the molecular diffusion coefficient of O_2 in water at a salinity of 36 and a temperature of 26 °C and δC/δz is the O_2 concentration gradient in the DBL. Dark respiration was estimated as the O_2 flux in darkness, while net photosynthesis was estimated by the O_2 flux in light. Gross photosynthesis measurements were performed at the symbiolite surface using the light-dark shift technique (Revsbech and Jørgensen, 1983) and data were recorded with a conventional strip-chart recorder with a rapid time response (BD25, Kipp & Zonen). Total areal rates of gross photosynthesis in symbiolites were estimated by integrating the volumetric photosynthetic rate over 100 μm (Revsbech and Jørgensen, 1983), i.e. the spatial resolution of the light-dark shift technique and the thickness of the symbiolites (Frommlet *et al.*, 2015). This calculation further assumes that photosynthesis is uniformly distributed with depth. To calculate the maximal photosynthesis rate, P_{max}, the light use efficiency (α) and the light acclimation index, E_k (μmol photons m^{-2} s^{-1}), the

measured photosynthesis irradiance (P-E) curves were fitted to an inverted exponential model by Webb *et al.* (1974):

$$P_G(E) = P_{\max}\left(1 - \exp^{\frac{-\alpha E}{P_{\max}}}\right).$$

E_k was calculated as P_{max}/α. Light respiration was calculated as the difference between areal gross and net photosynthesis.

Calcium concentration profiles were measured to investigate symbiolite calcification under flow conditions. To test whether calcification is induced by photosynthesis, we inhibited photosynthesis using the specific inhibitor of photosystem II, dichlorophenyldimethylurea (DCMU) (Bishop, 1958). DCMU was dissolved in ethanol and added to seawater to a final concentration of 1 μM (Al-Horani *et al.*, 2003). The ambient flow was increased to ensure effective mixing of DCMU in the experimental flow chamber. Calcium concentration profiles were measured before and 10 minutes after DCMU addition.

Quantification of precipitated $CaCO_3$ using LIX Ca^{2+} microsensors

For the determination of culture-specific, maximum amounts of precipitated $CaCO_3$, 32 *Symbiodinium* strains, covering a wide range of phylotypes, were inoculated at a ratio of 1:40 in 24-well plates for suspended cell cultures (Sarstedt) and were cultivated under standard conditions (see above) for 56 days. Based on previous work (Frommlet *et al.*, 20015) and the data presented in the present study, this culturing period generally ensures that calcifying cultures reach a post-calcifying stage. For the quantification of precipitated $CaCO_3$ in these cultures, we established a protocol that involved the removal of growth medium from the cultures, followed by the dissolution of the precipitated $CaCO_3$ in HCl and the determination of the calcium concentration in solution using the above Ca^{2+} microsensors. Empirical test showed that 0.5 mL of a 25 mM HCl (pH = 1.7) was sufficient to completely digest symbiolites within 35 min in well-calcified cultures of 2 mL (tested on strains 2, 12, 13, 23) (Figure 4 A, B). For the acid digestion, we removed the growth medium, added 0.5 mL of HCl (25 mM), and digested the samples for ~1h, followed by microscopic examination to confirm complete dissolution of symbiolites. Calcium concentrations were then directly measured using Ca^{2+} microsensors that were calibrated with 0, 0.002, 0.010, 0.050, 0.250, 0.500, 1, 10 and 20 mM $CaCl_2$ in 25 mM HCl. The performed tests to establish this protocol are further described below, in section 2.

2. Method development for the quantification of precipitated $CaCO_3$ in batch culture

Our initial approach to quantify *Symbiodinium*-driven calcification was to measure the associated reduction of calcium in the medium with Ca^{2+} microsensors, which is principally comparable to the established calcium anomaly technique (Chisholm and Gattuso, 1991; Gazeau *et al.*, 2015). Such a non-invasive approach would have allowed repeated measurements of calcification throughout the different phases of culture growth. However, trial experiments showed that (i) differences in electrode signal between calcified and uncalcified cultures were very small (<5 mV) and that (ii) measured calcium concentrations were much lower than expected seawater concentrations. The first problem was due to the high Ca^{2+} concentration of seawater of 10.6 mmol Kg^{-1} (Tyrell and Zeebe,

2004), which meant that concentration changes caused by $CaCO_3$ precipitation where in the upper, non-sensitive range of the Ca^{2+} electrode (Ammann *et al.*, 1987). The second problem, the apparent underestimation of actual calcium concentrations in algal cultures, was caused by the culture media, as a direct comparison of the electrode signal in ASP8A and f/2 medium with that in natural seawater showed the same artefacts (Supplementary Figure 2A, B). Monitoring of the electrode signal in seawater during the addition of different medium components showed how this underestimation arose (Supplementary Figure 2A). Basically, additions of vitamins and trace elements, monosodium phosphate and ammonium nitrate according to media recipes had no effect on electrode signal, but additions of sodium nitrate at concentrations as in f/2- and ASP8A-medium clearly lowered the electrode signal and thus interfered with calcium measurements in that the apparent calcium concentrations were lowered. This interference of $NaNO_3$ fundamentally contested direct calcium measurements in growth media such as f/2 and ASP8A as they are heavily supplemented with $NaNO_3$ as nitrogen source (Guillard, 1975). Alternative nitrate or ammonium salts may alleviate this problem because the interference is actually due to the sodium cation and not the nitrate anion (Ammann *et al.*, 1987). However, for the current study we did not attempt to further optimize this approach.

Instead we developed an alternative Ca^{2+} microsensor-based strategy that, on one hand, avoided the aforementioned problems but, on the other hand, was invasive and thus only allowed endpoint measurements of calcification. The approach involved the removal of the growth medium from the cultures, followed by the dissolution of the precipitated $CaCO_3$ in HCl and the measurement of resulting calcium concentrations using LIX Ca^{2+} microsensors (see Supplementary Materials and Methods for details). To evaluate this approach, we established that in the tested range of concentrations, electrode performance was not negatively affected by HCl (Supplementary Figure 3A, B), which confirmed that Ca^{2+} microsensors work in a wide pH range (de Beer *et al.*, 2000). Also, calcium standards prepared in 25 mM HCl (pH 1.7) with the commonly used $CaCl_2$ and, alternatively, with $CaCO_3$ to mimic symbiolite dissolution and the associated pH buffering effect, matched exceedingly well (Supplementary Figure 3C), showing that in the range of relevant concentrations there was no confounding effect of symbiolite dissolution and the associated pH buffering on electrode performance.

3. Extended discussion of variations in DIC limitation between experiments in batch cultures

The physicochemical conditions in batch cultures and how they related to the onset of symbiolite formation varied considerably between two sets of experiments. During a first set of experiments, cultures AV32 and Pk702 depleted DIC to such an extent that even a strong rise in pH did not cause a high Ω_{arag} and calcification. Considering that cultures were grown in tissue culture flasks with vented caps to support gas exchange, this DIC starvation during log-phase must be a common feature in *Symbiodinium* batch cultures. Yet, a second set of experiments with strains 74 and 203 showed that this is not always the case, as the drop in DIC in these cultures was far less pronounced, TA remained high, pH did not become as alkaline, and Ω_{arag} reached higher values compared to AV32 and Pk702 and co-varied with the pH dynamics. Strain-specific differences in growth rates could only explain the less dramatic decrease of DIC, if strains 74 and 203 would have lower growth rates than AV32 and Pk702. Evidently, not all strains of the diverse genus *Symbiodinium* grow equally fast but 74 and Pk702 are both fast-growing ITS2-type B1 strains (Figure 1B), and it is unlikely that any difference in growth rate between these sister strains would be large enough to explain the difference between the two experiments. Thus, instead of lower CO_2 consumption it is more likely that an additional DIC source caused the observed differences. The possibility of an external DIC source cannot be fully

excluded because CO_2 concentrations in the incubator were not measured, but there is no obvious reason why CO_2 concentrations in the incubator should have differed between experiments. Thus, an internal DIC source seems the more plausible explanation for the dampened DIC dynamics and the earlier onset of calcification during the second set of experiments. A higher abundance and/or metabolic activity of bacteria at the time of inoculation of the second experiment could be such an internal source, as increased bacterial respiration could have partially compensated the CO_2 assimilation by *Symbiodinium*. How such a higher abundance/activity of bacteria could arise is not clear but differences in the growth stage of pre-cultures at inoculation might be a decisive factor here.

4. Extended discussion of effect of culture disturbance on symbiolite formation

In addition to the negative effects of culture disturbance on calcification in AV32, PK702 and 203, which were likely due to the disruption of the biofilm structure and its microenvironment, our results also showed that in strain 74 disturbance had an overall calcification promoting effect (Figure 2; Supplementary Figure S4). In principle, several calcification-promoting and -inhibiting effects are conceivable. For instance, repeated opening of flasks and turbulent mixing could in principle have enhanced gas exchange, counteracting DIC depletion and promoting calcification. However, in the studied cultures this cannot have been a decisive factor because disturbance did not prevent the strong DIC depletion in AV32 and Pk702, and the less severe DIC depletion in 74 and 203 was evident in disturbed and undisturbed cultures alike (Figures 2 and 3). Culture disturbance might also influence the metabolism of *Symbiodinium* and/or the bacterial community positively by e.g. increasing nutrient uptake and mass transfer rates (e.g. review by Guasto *et al.*, 2012) or negatively by e.g. disturbing beneficial bacterial-bacterial or bacterial-algal associations (e.g. review by Seymour *et al.*, 2017). Data to best assess these potential effects are from AV32 and PK702 because, under both disturbed and undisturbed conditions these strains only calcified when physiology-driven changes had already relaxed. Hence, pH and carbonate system changes prior to calcification were purely physiology- and diffusion-driven. The fact that in these strains the photosynthesis-driven rise in pH and drop in DIC was slower in the disturbed cultures indicates that either algal photosynthesis was affected negatively or that bacterial respiration was affected positively by disturbance. Thus, exactly how physical disturbance affects the complex physiological interactions between *Symbiodinium* and bacteria remains unknown but on the level of culture as a whole it appears that effects of disturbance on physiology should have intensified the inhibitory effects on calcification. Finally, culture disturbance might also increase the number of coccoid vegetative cells, as *Symbiodinium* and other dinoflagellates react to hydromechanical shear stress with the shedding of their flagellae and the formation of ecdysal stages (Peters and Marrasé, 2000). As discussed above, a change from swimming zoospores to non-motile cells could cause a shift of photosynthetic activity from the bulk medium to the biofilm microenvironment, which could positively affect PCP. The calcification promoting effect of disturbance on strain 74 could thus indicate that zoospores of this strain are more sensitive to shear stress than zoospores of the other three strains.

5. Supplementary References

Al-Horani, F. A., Al-Moghrabi, S. M., and de Beer, D. (2003). The mechanism of calcification and its relation to photosynthesis and respiration in the scleractinian coral *Galaxea fascicularis*. *Mar. Biol.* 142, 419–426. doi:

Ammann, D., Bührer, T., Schefer, U., Müller, M., and Simon, W. (1987). Intracellular neutral carrier-based Ca^{2+} microelectrode with subnanomolar detection limit. *Pflugers Arch.* 409, 223–228. doi: 10.1007/BF00583469

Bishop, N. I. (1958). The influence of the herbicide, DCMU, on the oxygen-evolving system of photosynthesis. *Biochim. Biophys. Acta* 27, 205–206. doi: 10.1016/0006-3002(58)90313-5

Blank, R. J. (1987). Cell architecture of the dinoflagellate *Symbiodinium* sp. inhabiting the Hawaiian stony coral *Montipora verrucosa*. *Mar. Biol.* 94, 143–155. doi: 10.1007/BF00392906

Chisholm, J., and Gattuso, J. P. (1991). Validation of the alkalinity anomaly technique for investigating calcification of photosynthesis in coral reef communities. *Limnol. Oceangr.* 36, 1232–1239. doi: 10.4319/lo.1991.36.6.1232

de Beer, D., Glud, A., Epping, E., and Kühl, M. (1997). A fast-responding CO_2 microelectrode for profiling sediments, microbial mats, and biofilms. *Limnol. Oceangr.* 42, 1590–1600. doi: 10.4319/lo.1997.42.7.1590

de Beer, D., Kühl, M., Stambler, N., and Vaki, L. (2000). A microsensor study of light enhanced Ca^{2+} uptake and photosynthesis in the reef-building hermatypic coral *Favia* sp. *Mar. Ecol. Prog. Ser.* 194, 75–85. doi: 10.3354/meps194075

Dickson, A. G., and Millero, F. J. (1987). A comparison of the equilibrium constants for the dissociation of carbonic acid in seawater media. *Deep-Sea Res.* 34, 1733–1743. doi: 10.1016/0198-0149(87)90021-5

Fitt, W. K., and Trench, R. K. (1983). The relation of diel patterns of cell division to diel patterns of motility in the symbiotic dinoflagellate *Symbiodinium microadriaticum* Freudenthal in culture. *New Phytol.* 94, 421–432. doi: 10.1111/j.1469-8137.1983.tb03456.x

Frommlet, J. C., and Iglesias-Rodríguez, M. D. (2008). Microsatellite genotyping of single cells of the dinoflagellate species *Lingulodinium polyedrum* (Dinophyceae): a novel approach for marine microbial population genetic studies. *J. Phycol.* 44, 1116–1125. doi: 10.1111/j.1529-8817.2008.00566.x

Frommlet, J. C., Sousa, M. L., Alves, A., Vieira, S. I., Suggett, D. J., and Serôdio, J. (2015a). Coral symbiotic algae calcify *ex hospite* in partnership with bacteria. *Proc. Natl. Acad. Sci. U.S.A.* 112, 6158–6163. doi: 10.1073/pnas.1420991112

Gazeau, F., Urbini, L., Cox, T. E., Alliouane, S., Gattuso, J. P. (2015). Comparison of the alkalinity and calcium anomaly techniques to estimate rates of net calcification. *Mar. Ecol. Prog. Ser.* 527, 1–12. doi: 10.3354/meps11287

Gran G. (1950). Determination of the equivalent point in potentiometric titrations. *Acta. Chem. Scand.* 4, 559–577. doi: 10.3891/acta.chem.scand.04-0559

Gran G. (1952). Determination of the equivalence point in potentiometric titrations. Part II. *The Analyst* 77, 661–671. doi: 10.1039/AN9527700661

Guasto, J. S., Rusconi, R. and Stocker, R. (2012). Fluid mechanics of planktonic microorganisms. *Annu. Rev. Fluid Mech.* **44**, 373–400. doi: 10.1146/annurev-fluid-120710-101156

Guillard, R. R. L. (1975). "Culture of phytoplankton for feeding marine invertebrates," in *Culture of marine invertebrate animals*, eds: W. L. Smith, and M. H. Chanley (New York: Plenum Press), 26–60. doi: doi.org/10.1007/978-1-4615-8714-9_3

Hall, T. A. (1999). BioEdit: a user-friendly biological sequence alignment editor and analysis program for Windows 95/98/NT. *Nucl. Acids Symp. Ser.* 41, 95–98.

Hansson, I. (1973). A new set of acidity constants for carbonic acid and boric acid in sea water. *Deep-Sea Res.* 20, 461–478. doi: 10.1016/0011-7471(73)90100-9

Jimenez, I. M., Kühl, M., Larkum, A. W. D., and Ralph, P. (2011). Effects of flow and colony morphology on the thermal boundary layer of corals. *J. Roy. Soc. Interface* 8, 1785–1795. doi: 10.1098/rsif.2011.0144

LaJeunesse, T. C., and Trench, R. K. (2000). The biogeography of two species of *Symbiodinium* (Freudenthal) inhabiting the intertidal anemone, *Anthopleura elegantissima* (Brandt). *Biol. Bull.* 199, 126–34. doi: 10.2307/1542872

Mehrbach, C., Culberson, C. H., Hawley, J. E., and Pytkowicz, R. M. (1973). Measurement of the apparent dissociation constants of carbonic acid in seawater at atmospheric pressure. *Limnol. Oceanogr.* 18, 897–907. doi: 10.4319/lo.1973.18.6.0897

Mucci, A. (1983). The solubility of calcite and aragonite in seawater at various salinities, temperatures, and one atmosphere total pressure. *Am. J. Sci.* 283, 780–799. doi: 10.2475/ajs.283.7.780

Peters, F., and Marrasé C. (2000). Effects of turbulence on plankton: an overview of experimental evidence and some theoretical consideration. *Mar. Ecol. Prog. Ser.* 205, 291–306. doi: 10.3354/meps205291

Pierrot, D., Lewis, E., and Wallace, D. W. R. (2006). MS Excel program developed for CO_2 system calculations, Tech. rep., Carbon dioxide information analysis center, Oak Ridge National Laboratory, US Department of Energy, Oak Ridge, Tennessee. doi: 10.3334/cdiac/otg.co2sys_xls_cdiac105a

Revsbech, N. P. (1989). An oxygen microsensor with a guard cathode. *Limnol. Oceangr.* 34, 474–478. doi: 10.4319/lo.1989.34.2.0474

Revsbech, N. P., and Jørgensen B. B. (1983). Photosynthesis of benthic microflora measured with high spatial-resolution by the oxygen microprofile method - Capabilities and limitations of the method. *Limnol. Oceangr.* 28, 749–756. doi: 10.4319/lo.1983.28.4.0749

Rodhe, W., Vollenweider, R. A., and Nauwerck, A. (1958). "The primary production and standing crop of phytoplankton," in *Perspectives in Marine Biology,* ed. A. A. Buzzati-Traverso (Francisco: University of California Press), 299-322.

Rounds, S. A. (2012). "Alkalinity and acid neutralizing capacity (version 4.0)" in *National field manual for the collection of water-quality data*, eds. F. D. Wilde, and D. B. Radtke (U.S. Geological Survey Techniques of Water-Resources Investigations, Book 9, Chapter A6, Section 6.6, 45 p.

Santos, S. R., Taylor, D. J., and Coffroth, M. A. (2001). Genetic comparisons of freshly isolated versus cultured symbiotic dinoflagellates: implications for extrapolating to the intact symbiosis. *J. Phycol.* 37, 900–912. doi: 10.1046/j.1529-8817.2001.00194.x

Seymour, J. R., Amin, S. A., Raina, J. B., and Stocker, R. (2017). Zooming in on the phycosphere: the ecological interface for phytoplankton-bacteria relationships. *Nat. Microbiol.* 2:17065. doi: 10.1038/nmicrobiol.2017.65

Silva, A., Palma, S., Oliveira, P. B., and Moita, M. T. (2009). Composition and interannual variability of phytoplankton in a coastal upwelling region (Lisbon Bay, Portugal). *J. Sea Res.* 62, 238–249. doi: 10.1016/j.seares.2009.05.001

Tyrrell, T., and Zeebe, R. E. (2004). History of carbonate ion concentration over the last 100 million years. *Geochim. Cosmochim. Ac.* 68, 3521–3530. doi: 10.1016/j.gca.2004.02.018

Webb, W. L., Newton, M., and Starr, D. (1974). Carbon dioxide exchange of *Alnus rubra*. *Oecologia* 17, 281–291. doi: 10.1007/BF00345747

4. Supplementary Figures

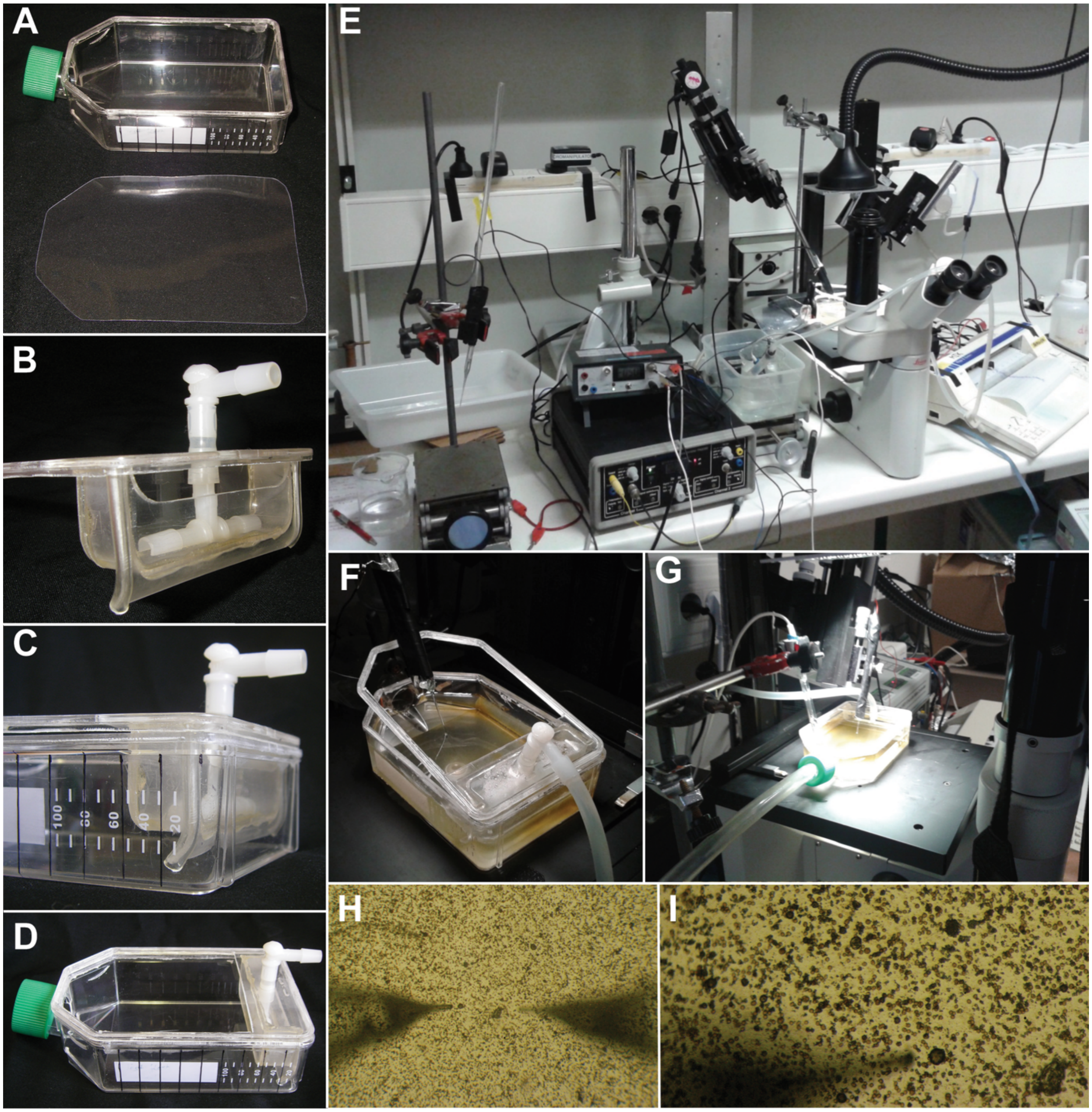

Supplementary Figure 1. Microsensor setup. (A) Cut-open tissue culture flask and removable lid. (B) Self-made laminar flow unit, built to fit into cut-open tissue culture flasks. (C, D) Assembled laminar flow chamber. (E) Overview of microsensor setup, showing inverted microscope base, light sources, micromanipulators, tubing and reservoir for temperature-controlled flow system, electronic equipment and strip-chart recorder for the determination of photosynthesis/respiration rates. (F) Insertion of laminar flow unit into a calcifying *Symbiodinium* culture, grown in a cut-open tissue culture flask (tubing for water circulation attached and microelectrode inserted). (G) Laminar flow chamber mounted on microscope stand and with water outflow attached. (H) Measuring tips of pH and Ca^{2+} microsensors approaching the surfaces of two adjacent symbiolites. (I) Close up of (H), showing how the tip of the microsensor on the left approaches a young symbiolite.

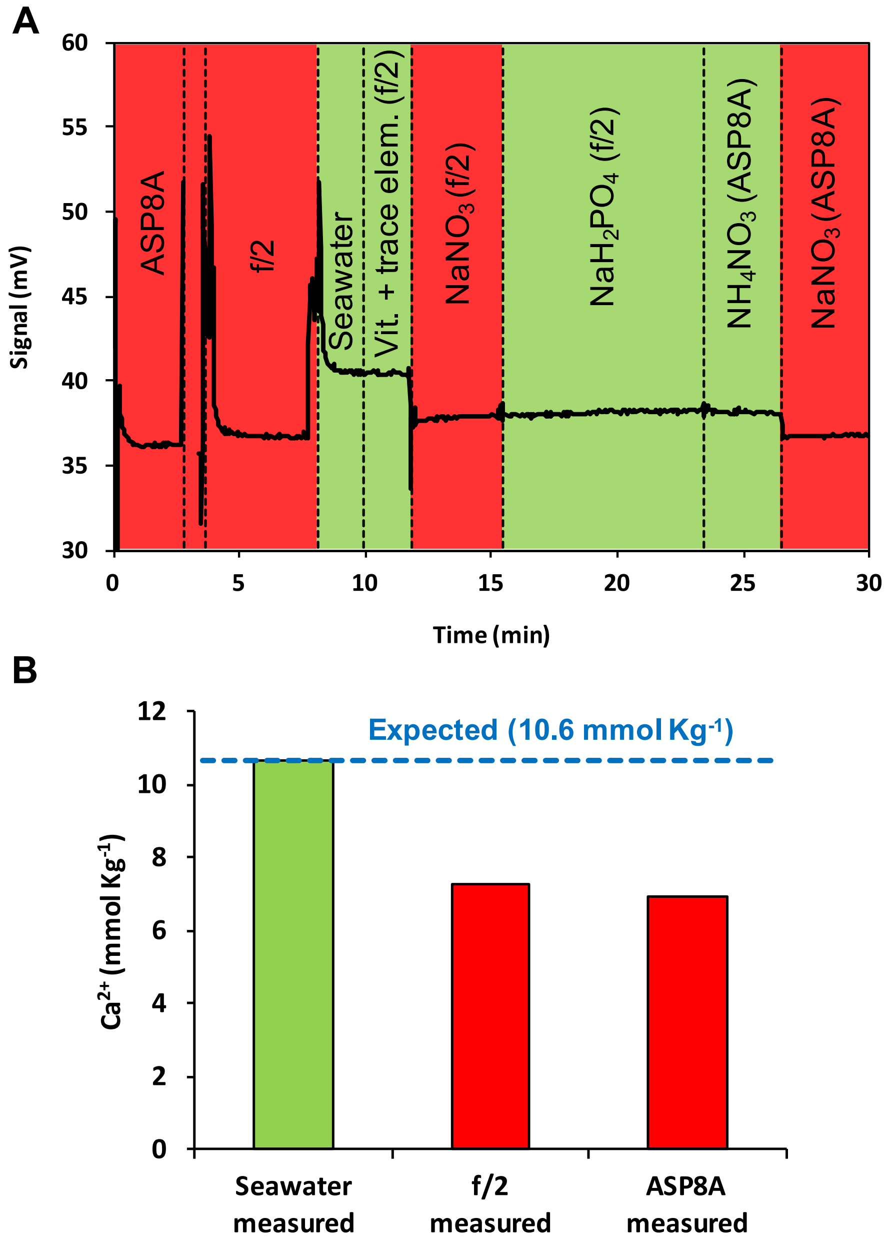

Supplementary Figure 2. Effect of ASP8A and f/2 medium components on Ca^{2+} microelectrode measurements. (A) Electrode signal trace in growth media (ASP8A and f/2), and in filtered natural seawater before and during the addition of different f/2 and ASP8A components according to media recipes (Guillard, 1975; Blank, 1987). Unexpectedly low electrode signals in media compared to seawater and artefacts caused by the addition of $NaNO_3$ according to the f/2 recipe (0.882 mM) and the ASP8A recipe (0.588 mM) are highlighted in red. Other tested media components did not change the electrode signal and are highlighted in green. Vit. + trace elem. = vitamins and trace elements. (B) Expected (blue dotted line; Tyrell and Zeebe, 2004) and measured Ca^{2+} concentrations in natural seawater and in f/2 and ASP8A medium based on raw signal trace in (A), showing how the addition of certain medium components to natural seawater leads to an underestimation of Ca^{2+} concentrations in the media.

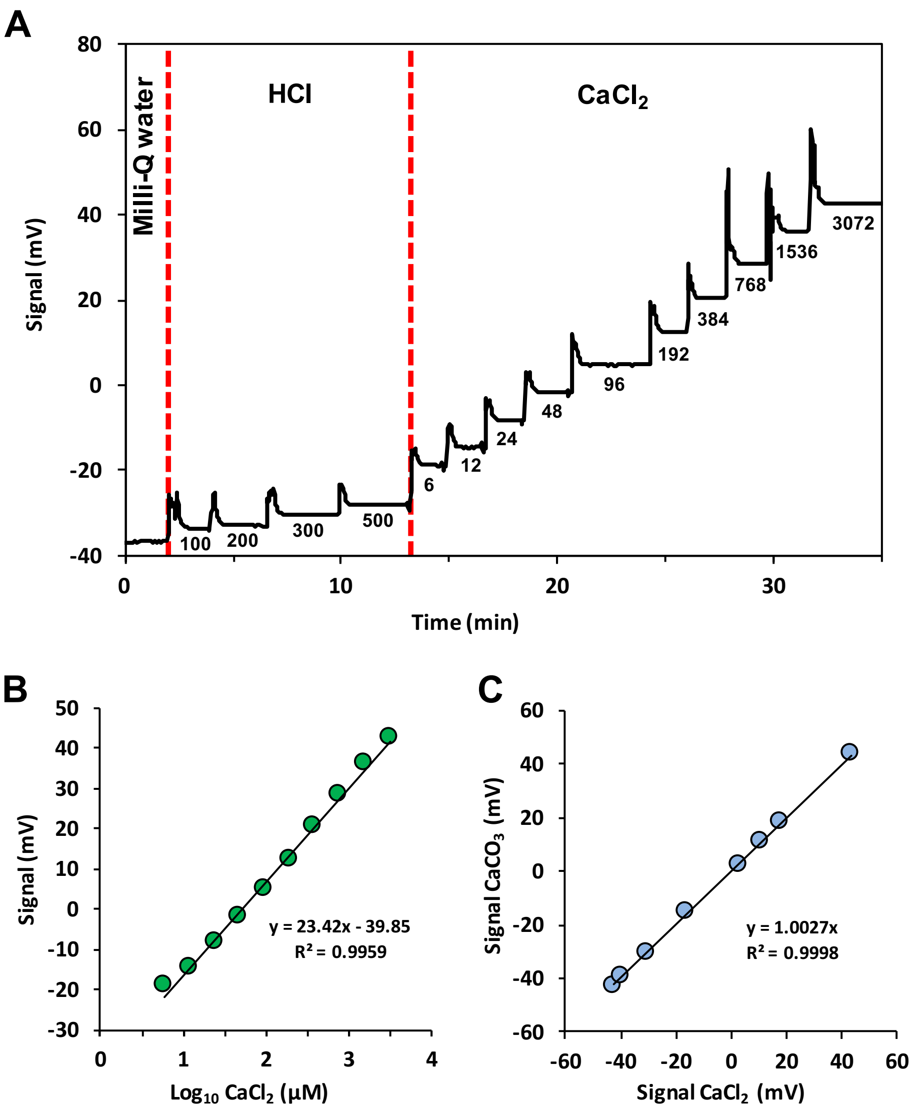

Supplementary Figure 3. Performance of Ca^{2+} microsensors in acidified Milli-Q water. (A) Electrode signal trace in Milli-Q water and during the stepwise addition of HCl to a final pH of 3, followed by stepwise addition of $CaCl_2$. Values below the signal trace are concentrations (µM) of HCl and $CaCl_2$, respectively. (B) Electrode signal in (A) plotted against Log_{10} of $CaCl_2$ concentrations in calibration standards. Linear response of the signal showed that the electrode performed well in acidified Milli-Q water. (C) Electrode signal of regular $CaCl_2$ calibration standards *vs.* $CaCO_3$ calibration standards (Ca^{2+} concentrations = 0, 0.002, 0.010, 0.050, 0.250, 0.500, 1, 10 mM) in 25 mM HCl, which was the HCl concentration used to dissolve symbiolites for analysis (see Supplementary Material and Methods). Linear correlation between standards showed that potential pH-buffering effects due to sample dissolution did not affect electrode performance.

4. Supplementary Tables

Supplementary Table 1. Onset of symbiolite formation in *Symbiodinium* batch culture.

Culture	Time (days)						
	0	4	8	13	23	29	42
61	-	-	-	+	+	+	+
362	-	-	-	-	+	+	+
370	-	-	-	-	-	+	+
23	-	-	-	-	+	+	+
89	-	-	-	+	+	+	+
97	-	-	-	+	+	+	+
104	-	-	-	-	-	+	+
130	-	-	-	-	-	+	+
185	-	-	-	+	+	+	+
PHMS TD1e	-	-	-	-	-	+	+
292	-	-	-	-	+	+	+
Culture X	-	-	+	+	+	+	+
PTA1	-	-	+	+	+	+	+
80	-	-	-	-	-	+	+
m. mirabilis	-	-	-	+	+	+	+
FLAp1	-	-	-	-	+	+	+
Pk708	-	-	-	+	+	+	+
AV32	-	-	-	-	+	+	+
99	-	-	-	+	+	+	+
105	-	-	-	+	+	+	+
107	-	-	-	+	+	+	+
108	-	-	-	+	+	+	+
154	-	-	-	-	+	+	+
2	-	-	-	+	+	+	+
12	-	-	-	+	+	+	+
13	-	-	-	+	+	+	+
64	-	-	-	+	+	+	+
74	-	-	-	-	+	+	+
Pk704	-	-	-	+	+	+	+
Pk706	-	-	-	+	+	+	+
146	-	-	-	+	+	+	+
147	-	-	-	-	-	+	+
M. capitata	-	-	-	-	+	+	+
351	-	-	-	+	+	+	+
Pk702	-	-	-	+	+	+	+
141	-	-	-	-	+	+	+
385	-	-	-	-	-	+	+
FLAp2	-	-	-	-	+	+	+
203	-	-	-	-	+	+	+
HHC1B	-	-	-	-	+	+	+
401	-	-	-	-	-	-	+
Ap31	-	-	-	+	+	+	+
383	-	-	+	+	+	+	+
135	-	-	-	-	+	+	+
133	-	-	-	-	+	+	+
**Number of calcifying cultures**	**0**	**0**	**3**	**23**	**37**	**44**	**45**
